# High diversity of root associated fungi in both alpine and arctic *Dryas octopetala*

**DOI:** 10.1186/1471-2229-10-244

**Published:** 2010-11-11

**Authors:** Marit Frederikke Markussen Bjorbækmo, Tor Carlsen, Anne Brysting, Trude Vrålstad, Klaus Høiland, Karl Inne Ugland, Jozsef Geml, Trond Schumacher, Håvard Kauserud

**Affiliations:** 1 Microbial Evolution Research Group (MERG), Department of Biology, University of Oslo, P.O. Box 1066 Blindern, N-0316 Oslo, Norway; 2 Centre for Ecological and Evolutionary Synthesis (CEES), Department of Biology, University of Oslo, P.O. Box 1066 Blindern, N-0316 Oslo, Norway; 3 Marine Biology, Department of Biology, University of Oslo, P.O. Box 1066 Blindern, N-0316 Oslo, Norway; 4 Kits van Waveren Foundation, Nationaal Herbarium Nederland, Universiteit Leiden, P.O. Box 9514, 2300 RA Leiden, Nederland

## Abstract

**Background:**

*Dryas octopetala *is a widespread dwarf shrub in alpine and arctic regions that forms ectomycorrhizal (ECM) symbiotic relationships with fungi. In this study we investigated the fungal communities associated with roots of *D. octopetala *in alpine sites in Norway and in the High Arctic on Svalbard, where we aimed to reveal whether the fungal diversity and species composition varied across the Alpine and Arctic regions. The internal transcribed spacer (ITS) region of nuclear ribosomal DNA was used to identify the fungal communities from bulk root samples obtained from 24 plants.

**Results:**

A total of 137 operational taxonomic units (OTUs) were detected (using 97% similarity cut off during sequence clustering) and well-known ECM genera such as *Cenococcum*, *Cortinarius, Hebeloma*, *Inocybe *and *Tomentella *occurred frequently. There was no decrease in fungal diversity with increasing latitude. The overall spatial heterogeneity was high, but a weak geographical structuring of the composition of OTUs in the root systems was observed. Calculated species accumulation curves did not level off.

**Conclusions:**

This study indicates that the diversity of fungi associated with *D. octopetala *does not decrease in high latitude arctic regions, which contrasts observations made in a wide spectrum of other organism groups. A high degree of patchiness was observed across root systems, but the fungal communities were nevertheless weakly spatially structured. Non-asymptotical species accumulation curves and the occurrence of a high number of singletons indicated that only a small fraction of the fungal diversity was detected.

## Background

The land area covered by arctic and alpine vegetation makes up roughly 11 million km^2^, an area comparable to that of boreal forests on the Northern and Southern Hemisphere. In most areas of the arctic and alpine zone, less than ten species constitute more than 90% of the vascular plant biomass [1]. The vast majority of plants form mycorrhizal relationships, a symbiosis considered favourable, especially for plants in nutrient-stressed situations [2]. Mycorrhiza may therefore be particularly beneficial in arctic ecosystems where low soil moisture and nutrient availability, low soil and air temperatures, and a short growing season limit plant growth and reproduction. It has been estimated that mycorrhizal fungi supply arctic plants with as much as 61-86% of the host plants nitrogen [3]. This implies that mycorrhizal fungi are the main nitrogen providers under the nitrogen-limited conditions in arctic tundra. Compared to the low plant diversity in arctic and alpine communities, the richness and heterogeneity of root-associated fungal communities is high [4].

Many factors and complex interactions influence the structure and composition of mycorrhizal communities [5]. For example, several studies have shown that mycorrhizal communities may change during ecosystem succession [6-10]. However, few studies have analysed how root associated fungal communities change along broader regional gradients, for example along latitudinal and longitudinal gradients. A widely recognised pattern in plant and animal ecology is the decrease in biological diversity with increasing altitude and latitude, but the underlying causes for this gradient are still poorly understood [e.g., [11-14]]. As pinpointed by Allen et al. [15], such patterns are almost unexplored in mycorrhizal fungi (but see [16]).

Ectomycorrhiza (ECM) is most common on woody perennial plants. In heath and tundra areas of arctic and alpine environments the long-lived dwarf shrub *Dryas octopetala *(Rosaceae) is of particular ecological significance [2,17]. *Dryas octopetala *have been found to be associated with many different ECM fruiting bodies [4,18,19]. Väre et al. [20] revealed by light microscopy that *D. octopetala *in western Spitsbergen (Svalbard) was symbiotic with 15 ECM fungal species. In a recent study by Ryberg et al. [21], the ECM diversity of *D. octopetala *and *Salix reticulata *in an alpine cliff ecosystem in northern Sweden was investigated using molecular methods. This survey documented a species rich fungal community dominated by *Cenococcum geophilum*, Thelephoraceae spp., *Cortinarius *spp., and Sebacinales spp. However, despite the ecological significance of fungi in arctic and alpine habitats, and that the documentation of fungal diversity in these climatic regions is of great importance due to global climatic changes, this is a sparsely investigated field.

The main aims of this study were to characterise the diversity of the fungal communities associated with roots of *D. octopetala *and to analyse the variation and change in the fungal communities, from alpine areas in the Central and Northern parts of Norway to the High Arctic in Svalbard. The following questions were asked: 1) Does the diversity of root associated fungi decrease towards arctic regions, as is the case for many other organism groups? 2) Is the fungal species composition different in arctic regions compared to more southern areas?

The fungal diversity was analysed by cloning and sequencing of ITS fragments from pooled root samples, and DNA similarity searches against UNITE [22] and GenBank [23]. In addition, a collection of ITS reference sequences were obtained from basidiocarps collected in arctic regions in order to improve the reference sequence library.

## Methods

### Material

**Figure 1:**
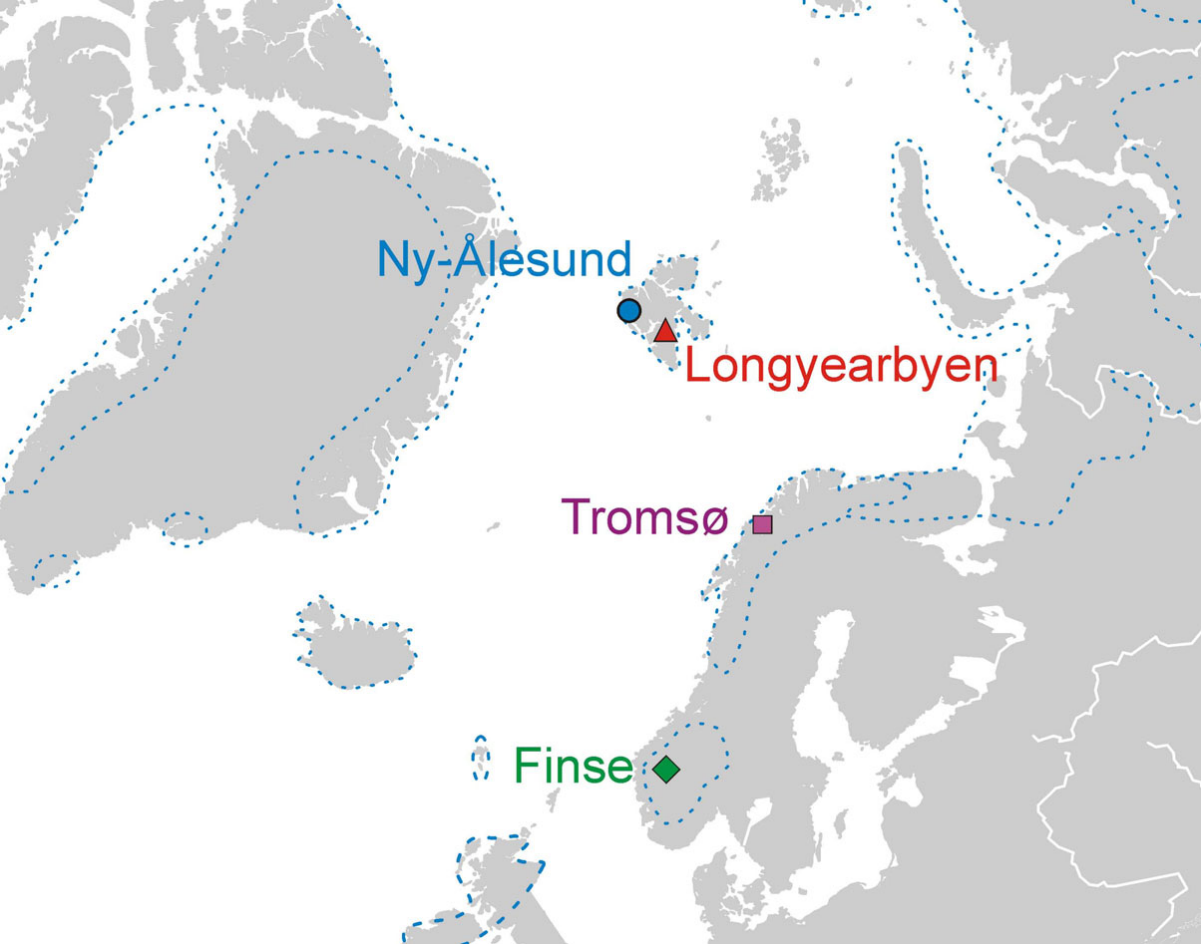
**Main localities**. Map indicating the main localities in Norway and Svalbard where *Dryas octopetala *root systems were sampled. Each main locality comprised three sub-localities separated by at least 1 km. The dotted line outlines the distribution area of *Dryas *in the North Atlantic region.

Twenty-four *Dryas octopetala *plants from four main localities situated on mainland Norway and Svalbard were sampled during the summers of 2006 and 2007 (Figure 1, Table 1). At each main locality, plants were sampled from three sub-localities (6 m × 6 m), each separated by approximately one kilometre. From each sub-locality, two plants with a well-defined and comparable spatial distribution of the root systems were excavated and stored in a cooling bag. Within 24 hours, the root systems were carefully rinsed under tap water, followed by distilled water (dsH_2_O). Approximately 40-50 mg of root fragments with living ECM root tips were detached from the cleaned root systems under a dissection microscope, and pooled in a 2 mL Eppendorf tube containing 1000 μl 2mM cetyltrimethylammonium bromide (CTAB) buffer. The samples were stored at -20°C until DNA extraction. Basidiocarps found within and in close proximity to the sub-localities were collected and determined to species. The 32 basidiocarps were dried at approximately 40°C and stored at room temperature until DNA-extraction. Supplementary samples from 157 specimens of arctic fungi deposited at the Natural History Museum, University of Oslo (O) were also included in the study. The list of basidiocarps is presented in Additional file 1.

**Table 1:** Localities.

Main locality	Sub-localities	Lat.	Long.	Elev.^1^	MJuly^2^	MJan^3^
Finse (Alpine)	F1: Kvannjolsnuten	60.608	7.549	1440	7.0°C	-10.1°C
	F2: Sandalsnuten	60.615	7.520	1480		
	F3: Jomfrunuten	60.604	7.519	1394		
Ny-Ålesund (Arctic)	N1: By Zeppelinfjellet	78.913	11.924	91	4.9°C	-13.9°C
	N2: West of Bayelva	78.934	11.835	20		
	N3: Sw of Knutsengheia	78.939	11.802	36		
Longyearbyen (Arctic)	S1: Endalen	78.189	15.781	34	5.9°C	-15.3°C
	S2: Longyeardalen	78.201	15.586	124		
	S3: Bjørndalen	78.231	15.333	30		
Tromsø (Alpine)	T1: Fløyfjellet	69.622	19.004	421	12.0°C	-4.5°C
	T2: Lyngen	69.694	20.778	180		
	T3: Lyngen, Steinfjellet	69.752	20.739	140		

Information about the localities where *Dryas octopetala *root systems were sampled.

^1^Elevation in metres above sea level. ^2^Mean July temperature at main localities. ^3^Mean January temperatures at main localities (temperature information from Norwegian Meteorological Institute).

### Molecular methods

Prior to DNA extraction, the pooled root samples were crushed with tungsten carbide beads for 2 min at 20Hz on a mixer mill (MM301, Retsch GmbH & Co, Haan, Germany). After two freeze-thaw steps (-80°C for 10 min) each sample was split into three Eppendorf tubes, of which one was used for further DNA extraction. DNA from the roots was first extracted with a 2mM CTAB miniprep method [24] using the modified protocol published by Gardes and Bruns [25]. The DNA was subsequently purified with the commercially available kit E.Z.N.A. Blood Kit (E.Z.N.A.^® ^Blood DNA Kit; Omega Bio-Tek, Doraville, GA) according to the manufacturer's protocol. DNA from the basidiocarps was extracted using the method above with minor modifications: DNA was resuspended in 60 μl dsH_2_O at the final step of extraction, and DNA templates were diluted 20 × before PCR amplification.

PCR amplification was performed using the fungal specific primers ITS1F and ITS4 [25,26]. The fidelity enzyme DyNAzyme EXT (Finnzymes Oy, Espoo, Finland) was employed according to the manufacturer's protocol. PCR was run in 25 μl reactions containing 16.5 μl of 10 × diluted template DNA and 8.5 μl reaction mix (2.5 μl EXT buffer, 2.5 μl dNTPs (2mM), 1.5 μl ITS1F primer (5 μM), 1.5 μl ITS4 primer (5 μM), and 0.5 μl DyNAzyme EXT). The PCR amplification conditions were as follows: 4 min at 94°C, followed by 35 cycles of 25 s at 94°C, 30 s at 52°C and 2 min at 72°C, followed by the final extension step for 10 min at 72°C before storage at 4°C. An elongated (i.e., 2 min) extension step was used to further minimise recombinant sequences (i.e. lower the frequency of incomplete ITS fragments present after each temperature cycle). PCR products (5 μl of each) were separated on agarose gels and stained with EtBr or SYBR_SAFE nucleic acid stain (Invitrogen Corporation, Carlsbad, CA) to visualise the PCR products prior to cloning. ITS fragments were amplified from the basidiocarps using the same PCR parameters.

The ITS fragments from the root samples were cloned with the TOPO TA Cloning kit (Invitrogen Corporation, Carlsbad, CA) using blue-white screening according to the manufacturer's manual. The clones were grown overnight in Luria-Bertani (LB) media amended with 50 μg/mL of ampicillin. From each plant root system, 32 bacterial colonies, representing cloned fragments, were subjected to PCR reactions with the vector primers T7 and M13R and using *c*. 0.5 μl of the bacterial suspension as template. The PCR amplification program was as follows: 5 min at 94°C followed by 30 cycles of 30 s at 94°C, 45 s at 52°C and 1.5 min at 72°C, followed by 7 min at 72°C before storage at 4°C. The PCR products were visualised on EtBr or SYBR_SAFE-stained gels and 24 randomly chosen cloned ITS fragments from each root system were sequenced, and visualised on an ABI 3730 DNA analyser (Applied Biosystems, Foster City). ITS amplicons from all reference basidiocarps were sequenced directly in both directions using primers ITS1F and ITS4, and visualised on an ABI 3730 DNA analyser.

All unique sequences as well as reference sequences have been accessioned in GenBank (see Additional files 1 and 2 for details).

### Sequence analyses

All sequence chromatograms were inspected manually and assembled in the program BioEdit Sequence Alignment Editor v.7.0.5 [27]. To control for potential chimaerical sequences, the alignment of 576 sequences was first inspected visually in order to detect sequences with recombination breakpoints, including non-coherent ITS1 and ITS2 types where ITS1 was identical to ITS1 of one genotype while ITS2, of the same sequence, was identical to ITS2 of another genotype. Identical ITS sequences detected in multiple root samples were considered authentic sequences while sequences detected only once (singletons) were controlled further by performing separate BLASTn searches [23] of the ITS1 and ITS2 regions. If there was consistency between the search results of ITS1 and ITS2 (i.e. ITS1 and ITS2 matched to the same species or genera), the ITS sequence was considered non-chimaerical. When discovered, chimaerical sequences were substituted with new (and controlled) sequences in order to obtain 24 non-chimaerical sequences from each root system.

Identical sequences were identified using ClustalW in BioEdit and a reduced dataset was constructed, including only sequences considered as unique. Artificial mutations introduced during the PCR process may occur using the cloning approach, which may lead to an overestimation of the molecular variation [28]. Therefore, single sequences with unique mutations but otherwise identical to other sequences amplified from the same root system, were not accepted as authentic unless more than two such mutations occurred in the same sequence [see [29] for a more thorough rationale for this approach]. In those cases where only two sequences amplified from the same root system were identical, except for one or two mutations, a consensus sequence was generated (using e.g. 'Y' when a 'C' occurred in one sequence and a 'T' in the other). Unique sequences were grouped into operational taxonomic units (OTUs) by performing a contig assembly in Sequencher v.4.1.4 (GeneCodes, Ann Arber, Michigan, USA) based on a 97% similarity criterion. All unique sequences in the reduced dataset were examined by BLASTn searches. In addition, local searches against the UNITE database [22] and against the 189 ITS basidiocarp sequences obtained in this study were performed.

ITS sequences with taxonomic affinity to the same genera were grouped into separate sub-alignments that also included the most similar accessions from GenBank or UNITE. Such alignments were established for the 11 most frequent and widespread taxonomic groups, mainly at the genus level, using ClustalW and manual adjustments. In addition, sequences from the reference basidiocarps were included in the sub-alignments.

OTUs were named based on the most similar accessions from GenBank, UNITE or basidiocarp sequences generated in this study. In cases with ≥97% similarity, the OTU was given a species name; in cases with <97% similarity, the OTU was given a genus name (e.g., *Tomentella *sp.1); in cases with <90% similarity, the OTU was given family or ordinal names (e.g., Cantharellales 1).

### Statistical analyses

Analysis of variance (ANOVA) was performed to reveal whether the average number of OTUs per rot systems differed between the main localities.

Phylogenetic analyses were conducted on the 11 sub-alignments using the maximum parsimony criterion in PAUP* (Phylogenetic Analysis Using Parsimony *and other methods) v.4.0 β10 [30]. A heuristic search with random stepwise addition of sequences, 10 replicates, and TBR (Tree Bisection and Reconnection) branch swapping was performed in order to improve the chances of finding the globally optimal solution (finding the most parsimonious trees). Gaps were treated as missing values. Trees were unrooted. All other settings were default. Strict consensus trees were created using TNT v.1.1 [31] for sub-alignments which resulted in more than one equally parsimonious tree. Collapsing rule was set to minimum length = 0. Jackknife branch support values were produced in TNT using random addition of sequences, 1000 search replicates, and cut-off value of 50%.

Detrended correspondence analysis (DCA) [32,33] and global non-metric multidimentional scaling (GNMDS) [34,35] ordinations were applied *in parallel *according to the procedure used by Økland et al. [36] to extract the main gradients in fungal OTU composition based on the presence/absence dataset of fungal OTUs in the 12 sub-localities, and in the 24 plant root systems. Congruent ordinations by the two methods were considered an indication that the main compositional gradients had been successfully recovered. The DCA calculations were performed in the *vegan *package v.1.9-13 [37] in R software, v.2.4.1 [38]. Detrending by segments and non-linear rescaling options were used to avoid arch and edge effects of correspondence analysis (CA) ordination [39]. The DCA ordination axes were scaled in standard deviation (S.D.) units. GNMDS were run using R software v.2.4.1, including packages *vegan *v.1.9-13 and *MASS*, using functions *vegdist*, *initMDS*, *isoMDS*, and *postMDS*, with options: dimensionality = 2; dissimilarity measure = percentage dissimilarity (Bray-Curtis) which with qualitative data reduces to Sørensen's index of dissimilarity [40], standardised by division with species maxima; minimum number of starting configurations = 100, of which one was the DCA; maximum number of iterations = 1000; stress reductions ratio for stopping iteration procedure = 0.99999. Solutions were not accepted unless reached from at least two different starting configurations. The degree of correspondence between axes obtained by DCA and GNMDS was tested by Kendall's rank correlation coefficients between scores along the first two DCA axes and the two GNMDS axes. All ordination diagrams were made by ArcView [41].

Species-accumulation curves and estimates of total OTU richness (OTU richness is hereafter referred to as species richness) of fungi associated with *D. octopetala *within the main localities, as well as in the entire study area, were calculated as proposed by Ugland et al. [42]. Traditional methods [e.g., [43-45]] for calculations of total species richness based on extrapolations from species-accumulation curves tend to underestimate species richness, because the addition of new samples normally leads to a vertical displacement of the species-accumulation curve [42]. Unlike the traditional methods, the method developed by Ugland et al. [42] and Ugland and Gray [46] recognises that heterogeneity in species richness can occur within sub-areas sampled and that this may have important consequences for the estimation of species richness. To estimate species richness in larger areas (i.e., more root systems in this case) than what has been sampled, this method [42] takes account of the spatial relationship between samples by dividing the sampled area into sub-areas (i.e., root systems). First a species-accumulation curve is obtained for randomised samples of all the single sub-areas, where the root systems were defined as sub-areas for estimating species richness within localities. Then the species-accumulation curve for all combinations of two sub-areas is calculated and the procedure is repeated for all sub-areas. It is the rate of increase of these new (and subsequent) species-accumulation curves as more sub-areas are combined that leads to the best estimate of total species (T-S). Thus, from the terminal points of the sub-area plots in the species-accumulation curves, a new T-S curve is obtained. The T-S curve can then be extrapolated to yield an estimate of the probable total number of species (OTUs) in the area. A bootstrapping method, sampling with replacement and re-sampling 100 times, was employed to judge the strength of support for the T-S estimates. Thus, by randomising the T-S estimates, variance measures were achieved.

## Results

### Sequence data

A total of 576 non-chimaerical ITS sequences were obtained, 24 from each of the 24 analysed root systems. Five additional sequences, 0.8% of all obtained sequences, were classified as chimaerical and omitted from further analyses. The 576 sequences represented 264 unique ITS genotypes of which 17 were detected in two or three independent root systems. These genotypes were grouped into 137 OTUs based on a 97% similarity cut-off level. A high proportion of the OTUs (80.4%) were detected in a single root system only, while 9.4% and 3.6% were detected in two and three root systems, respectively. A total of 119 of the 264 unique ITS sequences had 97% sequence similarity or higher to a reference sequence with known taxonomic affinity. Most sequences (81.9%) had best matches against GenBank accessions, 8.0% against UNITE accessions and 10.1% against the collection of reference sequences obtained in this study. The best matches in GenBank/UNITE of all unique sequences are listed in Additional file 2 and a list of all detected OTUs is presented in Additional file 3.

### Taxonomic distribution

**Figure 2:**
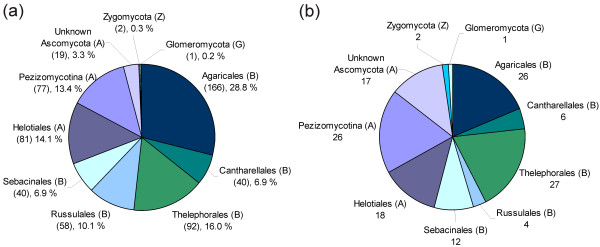
**Taxonomic coverage**. The taxonomic distribution of (a) the 576 analysed environmental ITS sequences, and (b) the taxonomic distribution of the 137 Operational Taxonomic Units (OTUs), where each OTU includes sequences that group together according to the 97% similarity criterion. Basidiomycota (B), Ascomycota (A), Zygomycota (Z), and Glomeromycota (G).

Based on identification by most similar reference sequences, 68.8% of the 576 sequences belonged to Basidiomycota, 30.7% to Ascomycota, 0.35% (two sequences) to Zygomycota, and 0.17% (one sequence) to Glomeromycota (Figure 2a). The corresponding numbers of OTUs in the four phyla were 75, 59, 2 and 1, respectively (Figure 2b). As shown in Figure 2a, Agaricales was the most commonly detected order (28.8% of the 576 sequences), followed by Thelephorales (16.0%), Helotiales (14.1%), and Russulales (10.1%). The corresponding distributions of OTUs in the various orders are presented in Figure 2b. ECM genera such as *Hebeloma*, *Cortinarius*, *Tomentella*, and *Inocybe *were frequently observed in the root systems of *D. octopetala*. It is noteworthy that the dominance of basidiomycetes over ascomycetes was much greater in terms of sequences (Figure 2a) than in terms of OTUs (Figure 2b). This is particularly striking when considering the 'Unknown Ascomycota' group with 19 sequences representing 15 separate OTUs. In contrast, the basidiomycete order Agaricales included 166 sequences that grouped into 26 different OTUs.

The 20 most frequently detected OTUs and their distribution across the four main localities are listed in Table 2. A high number of the sequences (92) had taxonomic affinity to Thelephorales, and this order also included most OTUs (27). The two most widespread OTUs had high sequence similarity (>99%) to accessions of *Cenococcum geophilum *and *Phialocephala fortinii *(Table 2). Two OTUs, both having best matches to *Cadophora finlandia *(98% similarity), were also widespread (Table 2). The OTU '*Cadophora finlandia *1' was detected in samples from the above-mentioned four main localities, while the OTU '*Cadophora finlandia *2' was represented in samples from one sub-locality in Tromsø and in all three Longyearbyen sub-localities. Another widespread OTU shared 99% similarity to reference sequences of both *Cortinarius *aff. *inconspicuus *and *C*. aff. *diasemospermus*.

**Table 2:** The most frequent OTUs.

OTU^1^	Taxonomic affinity	SS^2^	#C^3^	#RS (#ML)^4^	Sub-localities^5^
6	*Cenococcum geophilum*	100%	34	11 (4)	F1,F2,F3,N2,S3,T1,T2
4	*Phialocephala fortinii*	100%	34	10 (4)	F1,F3,N3,S2,S3,T1,T2,T3
28	*Cadophora finlandia*1	98%	13	7 (4)	F3,N1,N3,S2,S3,T2,T3
8	*Cortinarius *aff. *inconspicuus/diasemospermus*	99%	20	6 (3)	F1,F2,F3,N1,N2,S3
22	*Tomentella *sp.1	92%	8	4 (3)	F2,F3,N1,S1
67	*Caloplaca *sp.	91%	4	3 (3)	N3,S3,T1
80	*Cadophora finlandia *2	98%	12	5 (2)	S1,S2,S3,T3
83	Mycenaceae 1	82%	36	5 (2)	S2,T1,T2,T3
33	Inocybaceae 1	82%	12	3 (2)	F3,N2
2	*Hebeloma *aff. *alpinum*	99%	11	3 (2)	F1,N1,N2
39	*Tomentella *sp.2	94%	9	3 (2)	N1,N3,S1
43	*Tomentella *sp.3	95%	13	2 (2)	N1,S1
61	*Cortinarius *aff. *polaris*	100%	13	2 (2)	N3,S3
30	*Tomentella *sp.7	95%	3	2 (2)	F3,S1
63	*Hymenoscyphus *sp.	91%	3	2 (2)	N3,S1
57	*Leohumicola *sp.	94%	2	2 (2)	N2,T1
76	*Russula delica*	100%	31	4 (1)	S1,S2,S3
48	*Cadophora *sp.	96%	5	4 (1)	N1,N2,N3
47	*Cortinarius *aff. *tenebricus*	100%	11	2 (1)	N1,N3
40	*Tomentella *sp.4	93%	4	2 (1)	N1,N2

The 20 most frequently detected OTUs and their distribution across the five main localities.

^1^OTU number (see Additional files 2 and 3). ^2^Sequence similarity (SS) to best Blast match (GenBank/UNITE or own reference sequence). ^3^The number of clones (C) observed for the respective OTU. ^4^Number of root systems (RS), and number of main localities (ML) in brackets. ^5^Main localities; Finse (F), Longyearbyen (S), Ny-Ålesund (N), and Tromsø (T). Sub-localities indicated by numbers 1-3.

Sub-alignments were constructed for the 11 most frequently detected genera, also including the most similar reference sequences from GenBank or UNITE, in addition to congeneric basidiocarp reference sequences. The resulting phylogenetic trees are presented in Additional file 4, and show that many OTUs had a distinct geographic distribution.

### Diversity and composition of OTUs

**Figure 3:**
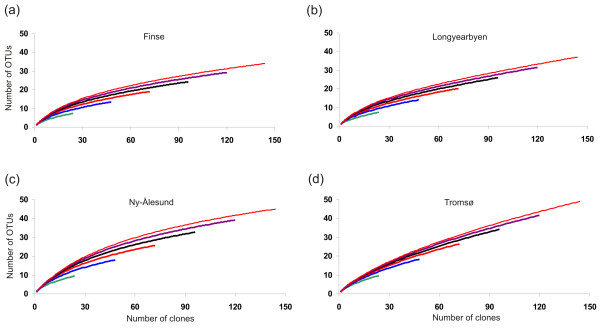
**Species-accumulation curves**. Species-accumulation curves for the separate main localities (a-d), where the number of Operational Taxonomic Units (OTUs) are plotted against number of root systems (i.e., the 24 clones from each of the root systems were grouped and defined as sub-areas). The various curves in the each diagram represent independent accumulation curves calculated with different number of root systems (24 clones from each). The end points of each curve can be used for extrapolation about total species richness (see Additional file 6), producing more realistic estimates than obtained by other non-parametrical methods.

The species-accumulation curves for the four main localities showed no sign of reaching an asymptote, nor did they show any latitudinal trend in richness of OTUs (Figure 3). Most OTUs were observed in Tromsø (49 OTUs), followed by Ny-Ålesund (45), Longyearbyen (37), and Finse (34). The average number of OTUs per root system was 8.2 in Tromsø followed by 7.5 in Ny-Ålesund, 6.2 in Longyearbyen, and 5.6 at Finse, which is a non-significant difference (ANOVA, p > 0.05). The extrapolated total species (T-S) curves further demonstrated a high degree of heterogeneity in the fungal communities, as none of them reached a plateau (Additional files 5 and 6). The estimated total richness of fungal OTUs, with 'sampling area' extrapolated to 10^6 ^root systems (24 clones from each) and randomisation by bootstrapping, resulted in estimation of 226 (± 32) OTUs to occur in the Tromsø main locality, 229 (± 28) in Ny-Ålesund, 177 (± 28) in Longyearbyen, and 169 (± 22) in Finse.

**Figure 4:**
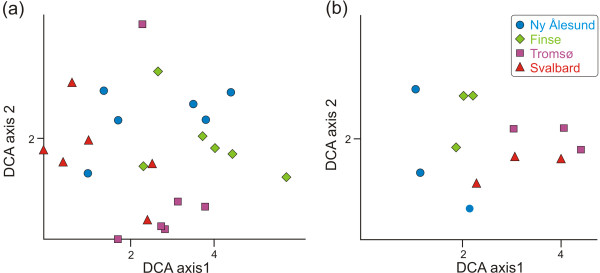
**Detrended Correspondence Analysis (DCA)**. DCA plots based on similarity in composition of OTUs in (a) the 24 *Dryas octopetala *root systems, and (b) the 12 sub-localities, where the two root systems from each sub-locality are grouped together. In plot (a) the plant root systems cluster independent from their geographic origin. However, in plot (b) the sub-localities cluster together, demonstrating that the ECM community of *D. octopetala *is geographically structured at this geographic scale. In plot (a) the gradient length of DCA axis 1 (DCA1) is 5.60 standard deviation (S.D.) units, and the length of DCA axis 2 (DCA2) is 4.22 S.D. units. The eigenvalue of DCA1 is 0.663, and the eigenvalue of DCA2 is 0.593. In plot (b) the gradient length of DCA1 is 3.26 S.D. units, and DCA2 is 2.35 S.D. units. The eigenvalue of DCA1 is 0.541, and 0.380 of DCA2.

Overall, the GNMDS ordination axes were strongly correlated with the corresponding DCA axes and the GNMDS ordination diagrams (data not shown) were visually similar to the DCA diagrams. Thus, only the DCA ordination results are presented (Figure 4), which have the advantage over GNMDS that the axes are scaled in standard deviation (S.D) units [e.g. [39]]. In the DCA analysis based on similarity in composition of OTUs of the various plant root systems (Figure 4a), only a weak geographical structuring can be observed along the two first DCA axes. The DCA analysis based on sub-localities (i.e. where incidence data from two plant root systems were pooled) displayed more distinct geographical structuring, which can be observed along the first two DCA axes (Figure 4b). It is noteworthy that the sub-localities did not cluster according to a latitudinal gradient neither along DCA axis 1 nor along DCA axis 2.

**Figure 5:**
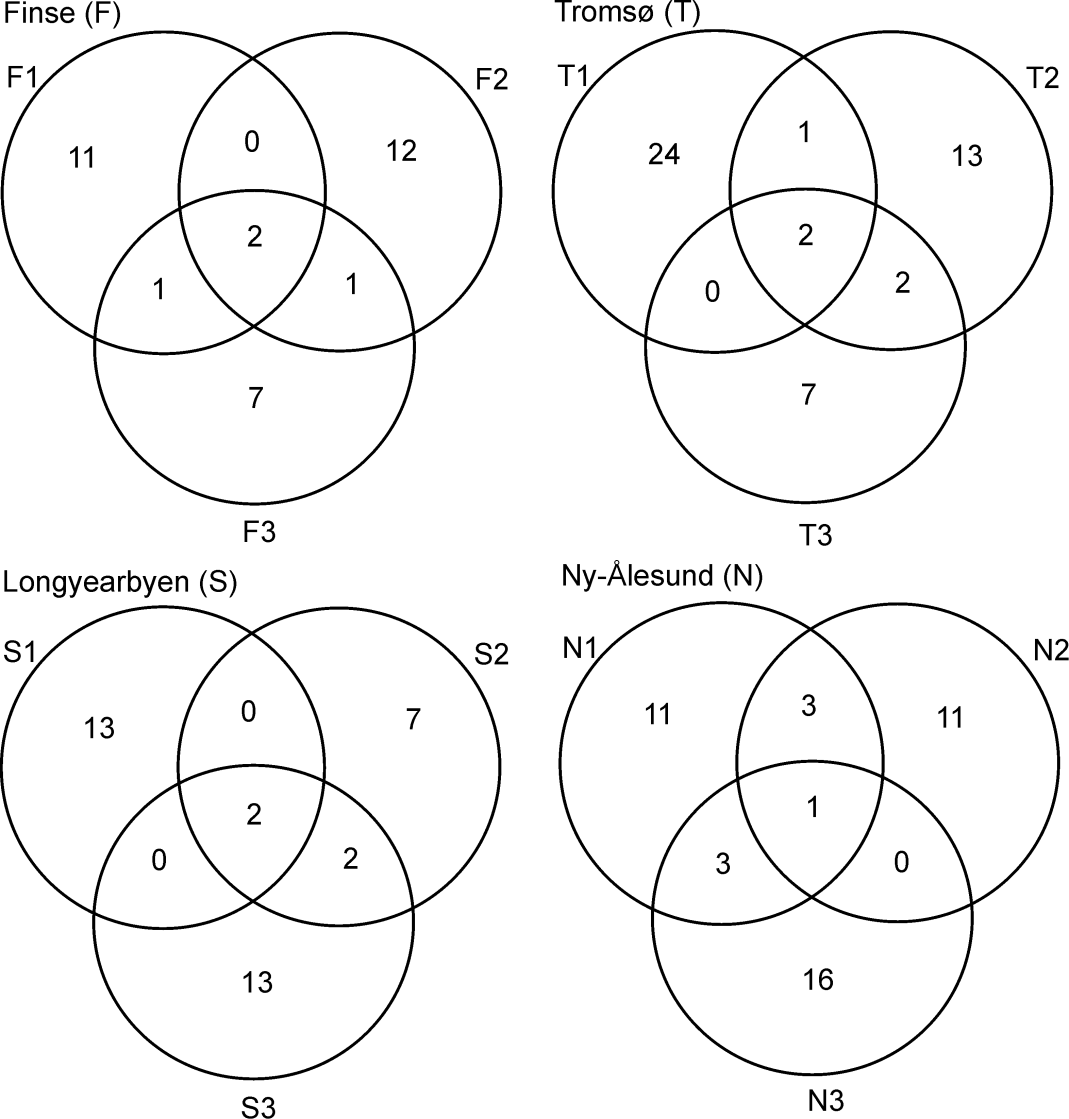
**Venn diagrams**. Venn diagrams demonstrating the number of shared OTUs between the three sub-localities from each main locality.

The average number of shared OTUs between root systems was 1.17 within sub-localities, 1.06 between sub-localities, and 0.65 between main localities. The Venn diagrams in Figure 5 displays the low number of overlapping OTUs between the three sub-localities from each main locality.

## Discussion

### Observed diversity

Using a 97% cut-off during sequence clustering, a total of 137 OTUs were detected in the 24 root systems, which is significantly higher than what have been observed in earlier diversity surveys of root associated fungi in arctic-alpine plants and environments [20,47-52]. Harrington and Mitchell [53-55] conducted several studies of ECM fungi associated with *D. octopetala *populations in western Ireland by morphotyping root tips and subsequent ITS sequencing. They detected 34 ECM morphotypes, of which 11 were identified to species or genus. Using ITS2 and similar sequence clustering conditions as in our study, Ryberg et al [21] observed 74 OTUs in 48 root systems of *D. octoptala *from Northern Sweden. The higher diversity detected in our study may be ascribed to the different sampling and molecular methodologies applied. For example, the applied clone based approach probably is more efficient in detecting the diverse array of fungi associated with the root systems compared to root tip morphotyping with subsequent sequencing. The latter approach often fails to identify many of the fungal symbionts [56,57]. The observed non-asymptotical accumulation curves and the long tail of rare genotypes observed in our study demonstrate that the 24 sequences generated from each root system did not cover the entire diversity of root associated fungi of *D. octopetala*.

### Taxonomic coverage

Members of Basidiomycota were more frequent than those of Ascomycota in the root systems of *D. octopetala*, in particular in terms of the number of sequences. This difference was not as pronounced when counting the involved OTUs (Figure 2a,b). This corroborates earlier observations that a small number of ECM species, in most cases basidiomycetes, are highly abundant and dominant [58]. Basidiomycetes are often represented by extensive fungal biomass in the form of thick ECM mantles and extraradical mycelium, while many ascomycetes may have thin mantles and a sparse amount of external mycelium [e.g., ECM of Cadophora finlandia; [59]], or they may be present as endophytes. This is in accordance with the most commonly detected taxonomic groups in this study being ECM genera of Basidiomycota that produce an extensive amount of external hyphae, such as *Hebeloma *[60], *Inocybe *[61], *Tomentella *[62], and *Cortinarius *[63].

A high number of ITS sequences and OTUs had taxonomical affinities to order Thelephorales. This fungal group was also one of the most frequently detected in a survey of ECM fungi associated with *D. octopetala *and *Salix reticulata *in Northern Sweden [21]. This suggests that these fungi are of special importance in the fungal communities associated with *D. octopetala *in arctic and alpine environments. The hyphal cell walls of the Thelephorales are generally melanised, which has been hypothesised to be an adaptation to resist attack from antagonistic fungi and fungivorous soil fauna, and to protect hyphae from extreme temperatures and drought. Melanised hyphae may also play a significant role in the fungus' persistence from year to year [64,65]. Interestingly, a high number of sequences with affinity to Thelephorales were observed in samples from the northernmost localities (Longyearbyen and Ny-Ålesund on Svalbard). It is noteworthy that most of the matches against reference sequences were quite low (typically 91-95% identity, with query coverage 99-100%), indicating that the arctic-alpine Thelephorales represent an unexplored group of fungi.

A striking feature emerging from studies of a wide array of plants that are growing in arctic and alpine environments is the extensive occurrence of dark septate endophytic (DSE) fungi in their roots [e.g., [2,20,52,66-68]], which are also characterized by melanised cell walls. DSE fungi were among the most frequently detected ascomycetes in our study. These included *Phialocephala fortinii*, *Cadophora finlandia*, and *Leptodontidium orchidicola*, fungi that might have a mycorrhizal and beneficial function in these habitats [67,69]. *Phialocephala fortinii *was detected in all main localities (10 of the 24 root systems) and has been recognized as one of the most abundant DSE in roots of conifers and ericaceous plants in heathlands, forests, and alpine ecosystems [e.g., [52,70-74]]. Two different OTUs with close affinity to *Cadophora finlandia *were observed. *Cadophora finlandia *forms characteristic melanised ECM morphotypes [59], and has frequently been observed in harsh habitats [e.g., on metal polluted or burnt sites; [74]].

Our study also corroborates the view that the *Cenococcum geophilum *complex, another melanised ECM fungus, is widespread and frequent in arctic and alpine environments [4,6,47-50]. This species was the most frequently encountered OTU, and was observed in about half of the root systems from all main localities.

The high occurrence and diversity of melanised fungi found in the present study indicate that they hold an important ecological function as associated with *D. octopetala *in arctic-alpine environments.

### Composition and distribution of OTUs

Even at a local scale (within sub-localities) there was little overlap in fungal OTUs across root systems, indicating high spatial heterogeneity. This finding is in agreement with earlier studies where community composition has been shown to be highly variable and patchily distributed at fine scales [75-77]. The ordination analysis of the 24 plant root systems (Figure 4a) further underlines the high degree of spatial heterogeneity, as little grouping of root systems according to locality was observed. Neither the species-accumulation curves, nor the extrapolated total species richness curves, showed any sign of reaching an asymptote, which also suggests a high spatial heterogeneity. A possible explanation for the high spatial heterogeneity is that plant root systems, as well as the fungal mycelium, are three dimensional, displaying a fractal-like geometric structure, and that the associated biotic factors such as soil microbes, and abiotic factors such as minerals, nutrient, and water supply, also usually vary at microscales in the soil, both spatially and temporally [2,5,58,78]. Due to the high complexity of the root system and the associated environmental factors, there are potentially a high number of micro-niches. This could explain the high fungal diversity and the high degree of spatial turnover in fungal communities observed at the different geographic levels. However, it must be emphasised that a higher sampling intensity is necessary to finally conclude on this matter.

There was no decrease in number of OTUs with increasing latitude, which contrasts the general pattern observed in other organisms groups [79] such as benthic marine invertebrates [80], birds [81,82], mammals [83,84], plants [85,86], and foliar fungal endophytes [87]. Arctic-alpine soils seem unexpectedly rich in diversity of microorganisms compared to their depauperate plant communities [4,15]. There are even some indications that microbial diversity in some cases is higher in the arctic than in boreal soils [88,89]. Hence, the belowground diversity of root-associated fungi may not follow the same latitudinal trend as the aboveground diversity.

In spite of the high heterogeneity and species turnover even at small scales, there seems to be a slight geographical structuring of the composition of fungal OTUs in the *D. octopetala *root systems at a larger geographical scale, as demonstrated by the ordination analysis of sub-localities (each including two analysed root systems; Figure 4b). In line with this, root systems within the same main localities and sub-localities had more OTUs in common compared to root systems compared across the main localities. The underlying cause for the geographic structuring at larger spatial scales is likely that some OTUs have a distinct biogeographical structure, as can be seen in some of the phylogenetic trees of selected taxonomic groups (Additional file 4). Some OTUs were only recorded from Svalbard (in multiple root systems), including several OTUs with affinity to Thelephorales. These OTUs (and many more, see Additional file 4) could be examples of fungi with a distinct arctic affiliation. There was no latitudinal trend in the ordination plot among the main localities, indicating that other factors than those associated with latitude structure the root associated fungal community of *D. octopetala *at a larger geographic scale. As highlighted by Bruns [5], almost 15 years ago, the factors which control and structure fungal diversity at a global, regional, and even single-root level, still remain a subject of debate. While overall diversity is thought to be important to ecosystem functioning, the functional significance of individual taxa is very poorly understood [77], but the high species richness of mycorrhiza in many ecosystems suggests a high level of functional heterogeneity may occur, even at the local scale [90].

## Conclusions

This study demonstrates that a phylogenetically diverse array of fungi is associated with roots of the arctic-alpine plant *Dryas octopetala*. Both the non-asymptotic species-accumulation curves, the disparity between observed and estimated species richness, and the fact that most OTUs were detected only once, suggest that the species richness is even higher than recorded here, and that many fungal species remain undetected. Noteworthy, we observed no decrease in fungal species richness in the Arctic. A weak spatial structuring of the composition of OTUs was observed, which would probably have been more pronounced with a higher sampling intensity.

## Authors' contributions

HK, MFMB and AB planned and coordinated the study. MFMB, AB, KH, JG and HK collected the study material and MFMB, TC, KIU, JG and HK conducted the molecular and statistical analyses. MFMB, TC and HK drafted the manuscript. All authors commented upon the manuscript and approved the final manuscript.

## Supplementary Material

Additional file 1 **List of basidiocarps used as reference sequences**. Click here for file

Additional file 2 **List of all unique sequences with their best matches in GenBank/UNITE/basidiocarp reference sequences**. Click here for file

Additional file 3 **List of all detected OTUs**. Click here for file

Additional file 4 **Phylogenetic trees of 11 prevalent taxonomic groups**. Click here for file

Additional file 5 **Observed and estimated number of OTUs**. Click here for file

Additional file 6 **Extrapolated total species (T-S) curves**. Click here for file

## References

[B1] Chapin FS Körner CH Chapin FS and CH Körner Patterns, causes, changes, and consequences of biodiversity in arctic and alpine ecosystems Arctic and alpine biodiversity 1995 Springer-Verlag Berlin Heidelberg 313 320

[B2] Smith SE Read DJ Mycorrhizal symbiosis 2008 Third UK, London: Academic Press

[B3] Hobbie JE Hobbie EA N-15 in symbiotic fungi and plants estimates nitrogen and carbon flux rates in Arctic tundra Ecology 2006 87 4 816 822 10.1890/0012-9658(2006)87[816:NISFAP]2.0.CO;2 16676524

[B4] Gardes M Dahlberg A Mycorrhizal diversity in arctic and alpine tundra: An open question New Phytologist 1996 133 1 147 157 10.1111/j.1469-8137.1996.tb04350.x

[B5] Bruns TD Thoughts on the processes that maintain local species-diversity of ectomycorrhizal fungi Plant and Soil 1995 170 1 63 73 10.1007/BF02183055

[B6] Trowbridge J Jumpponen A Fungal colonization of shrub willow roots at the forefront of a receding glacier Mycorrhiza 2004 14 5 283 293 10.1007/s00572-003-0264-3 14530929

[B7] Cazares E Trappe JM Jumpponen A Mycorrhiza-plant colonization patterns on a subalpine glacier forefront as a model system of primary succession Mycorrhiza 2005 15 6 405 416 10.1007/s00572-004-0342-1 15772815

[B8] Jumpponen A Soil fungal community assembly in a primary successional glacier forefront ecosystem as inferred from rDNA sequence analyses New Phytologist 2003 158 3 569 578 10.1046/j.1469-8137.2003.00767.x 36056507

[B9] Jumpponen A Trappe JM Cazares E Occurrence of ectomycorrhizal fungi on the forefront of retreating Lyman Glacier (Washington, USA) in relation to time since deglaciation Mycorrhiza 2002 12 1 43 49 10.1007/s00572-001-0152-7 11968946

[B10] Toljander JF Eberhardt U Toljander YK Paul LR Taylor AFS Species composition of an ectomycorrhizal fungal community along a local nutrient gradient in a boreal forest New Phytologist 2006 170 4 873 883 10.1111/j.1469-8137.2006.01718.x 16684245

[B11] Gaston KJ Global patterns in biodiversity Nature 2000 405 6783 220 227 10.1038/35012228 10821282

[B12] Pianka ER Latitudinal gradients in species diversity - a review of concepts American Naturalist 1966 100 910 33 46 10.1086/282398

[B13] Pianka ER Latitudinal gradients in species-diversity Trends in Ecology & Evolution 1989 4 8 223 223

[B14] Willig MR Kaufman DM Stevens RD Latitudinal gradients of biodiversity: Pattern, process, scale, and synthesis Annual Review of Ecology Evolution and Systematics 2003 34 273 309 10.1146/annurev.ecolsys.34.012103.144032

[B15] Allen EB Allen MF Helm DJ Trappe JM Molina R Rincon E Patterns and regulation of mycorrhizal plant and fungal diversity Plant and Soil 1995 170 47 62 10.1007/BF02183054

[B16] Olsson PA Eriksen BE Dahlberg A Colonization by arbuscular mycorrhizal and fine endophytic fungi in herbaceous vegetation in the Canadian High Arctic Canadian Journal of Botany-Revue Canadienne De Botanique 2004 82 11 1547 1556 10.1139/b04-111

[B17] Walker MD Walker DA Auerbach NA Plant communities of a tussock tundra landscape in the Brooks Range Foothills, Alaska Journal of Vegetation Science 1994 5 6 843 866 10.2307/3236198

[B18] Gulden G Torkelsen A-E Part 3. Fungi I. Basidiomycota: Agaricales, Gasteromycetales, Aphyllophorales, Exobasidiales, Dacrymycetales and Tremellales A catalogue of Svalbards plants, fungi, algae and cyanobacteria 1996 173 206

[B19] Høiland K Studies of ectomycorrhiza on Svalbard Agarica 1998 15 24/25 133 147

[B20] Väre H Vestberg M Eurola S Mycorrhiza and root-associated fungi in Spitsbergen Mycorrhiza 1992 1 3 93 104 10.1007/BF00203256

[B21] Ryberg M Larsson E Molau U Ectomycorrhizal Diversity on Dryas octopetala and Salix reticulata in an Alpine Cliff Ecosystem Arctic Antarctic and Alpine Research 2009 41 4 506 514 10.1657/1938-4246-41.4.506

[B22] Kõljalg U Larsson KH Abarenkov K Nilsson RH Alexander IJ Eberhardt U Erland S Hoiland K Kjoller R Larsson E Pennanen T Sen R Taylor AFS Tedersoo L Vralstad T Ursing BM UNITE: a database providing web-based methods for the molecular identification of ectomycorrhizal fungi New Phytologist 2005 166 3 1063 1068 10.1111/j.1469-8137.2005.01376.x 15869663

[B23] Altschul SF Madden TL Schaffer AA Zhang JH Zhang Z Miller W Lipman DJ Gapped BLAST and PSI-BLAST: a new generation of protein database search programs Nucleic Acids Research 1997 25 17 3389 3402 10.1093/nar/25.17.3389 9254694 PMC146917

[B24] Murray MG Thompson WF Rapid isolation of high molecular-weight plant DNA Nucleic Acids Research 1980 8 19 4321 4325 10.1093/nar/8.19.4321 7433111 PMC324241

[B25] Gardes M Bruns TD ITS primers with enhanced specificity for basidiomycetes - application to the identification of mycorrhizae and rusts Molecular Ecology 1993 2 2 113 118 10.1111/j.1365-294X.1993.tb00005.x 8180733

[B26] White TJ Bruns T Lee S Taylor J Innis MA, et al Amplification and direct sequencing of fungal ribosomal RNA genes for phylogenetics PCR protocols: a guide to methods and applications 1990 Academic Press: San Diego, CA, USA 315 322

[B27] Hall TA BioEdit: a user-friendly biological sequence alignment editor and analysis program for Windows 95/98/NT Nucleic Acids Symposium Series 1999 41 95 9810780396

[B28] Thornhill DJ Lajeunesse TC Santos SR Measuring rDNA diversity in eukaryotic microbial systems: how intragenomic variation, pseudogenes, and PCR artifacts confound biodiversity estimates Molecular Ecology 2007 16 24 5326 5340 10.1111/j.1365-294X.2007.03576.x 17995924

[B29] Popp M Oxelman B Inferring the history of the polyploid *Silene aegaea *(Caryophyllaceae) using plastid and homoeologous nuclear DNA sequences Molecular Phylogenetics and Evolution 2001 20 3 474 481 10.1006/mpev.2001.0977 11527472

[B30] Swofford DL PAUP* Phylogenetic Analysis Using Parsimony (and other methods) 2002 Sinauer Associates: Sunderland, MA

[B31] Goloboff PA Farris JS Nixon KC TNT, a free program for phylogenetic analysis Cladistics 2008 24 5 774 786 10.1111/j.1096-0031.2008.00217.x

[B32] Hill MO Gauch HG Detrended correspondence analysis: an improved ordination technique Vegetatio 1980 42 1-3 47 58 10.1007/BF00048870

[B33] Hill MO DECORANA. A fortran program for detrended correspondence analysis and reciprocal averaging 1979 Cornell University, Ithaca: New York

[B34] Kruskal JB Multidimensional scaling by optimizing goodness of fit to a nonmetric hypothesis Psychometrika 1964 29 1 1 27 10.1007/BF02289565

[B35] Kruskal JB Nonmetric multidimensional scaling: a numerical method Psychometrika 1964 29 2 115 129 10.1007/BF02289694

[B36] Økland T Bakkestuen V Okland RH Eilertsen O Changes in forest understorey vegetation in Norway related to long-term soil acidification and climatic change Journal of Vegetation Science 2004 15 4 437 448 10.1111/j.1654-1103.2004.tb02282.x

[B37] Oksanen J Kindt R Legendre P O'Hara B Package "Vegan" University of Oulu, Oulu 2007

[B38] R-Development-Core-Team R: A language and environment for statistical computing R Foundation for Statistical Computing, Vienna, Austria 2006 http://www.R-project.org

[B39] Økland RH Vegetation ecology: theory, methods and applications with reference to Fennoscandia Sommerfeltia 1990 Suppl. 1 1 233

[B40] Sørensen T A method of establishing groups of equal amplitude in plant society based on similarity of species content Det Kongelige Danske Videnskap Selskap 1948 5 1 34

[B41] ESRI Inc ArcView GIS 3.3 1999 Environmental Systems Research Institute Inc., Redlands, CA

[B42] Ugland KI Gray JS Ellingsen KE The species-accumulation curve and estimation of species richness Journal of Animal Ecology 2003 72 5 888 897 10.1046/j.1365-2656.2003.00748.x

[B43] Colwell RK Coddington JA Estimating terrestrial biodiversity through extrapolation Philosophical Transactions of the Royal Society of London Series B-Biological Sciences 1994 345 1311 101 118 10.1098/rstb.1994.0091 7972351

[B44] Colwell RK Mao CX Chang J Interpolating, extrapolating, and comparing incidence-based species accumulation curves Ecology 2004 85 10 2717 2727 10.1890/03-0557

[B45] Chao A Nonparametric-estimation of the number of classes in a population Scandinavian Journal of Statistics 1984 11 4 265 270

[B46] Ugland KI Gray JS Estimation of species richness: analysis of the methods developed by Chao and Karakassis Marine Ecology-Progress Series 2004 284 1 8 10.3354/meps284001

[B47] Muhlmann O Peintner U Ectomycorrhiza of *Kobresia myosuroides *at a primary successional glacier forefront Mycorrhiza 2008 18 6-7 355 362 10.1007/s00572-008-0188-z 18679725

[B48] Muhlmann O Peintner U Mycobionts of *Salix herbacea *on a glacier forefront in the Austrian Alps Mycorrhiza 2008 18 4 171 180 10.1007/s00572-008-0169-2 18365257

[B49] Muhlmann O Bacher M Peintner U *Polygonum viviparum *mycobionts on an alpine primary successional glacier forefront Mycorrhiza 2008 18 2 87 95 10.1007/s00572-007-0156-z 18064497

[B50] Sønstebø JH Molecular ecology of ectomycorrhizal fungi on *Bistorta vivipara *(L.) Gray in four alpine tundra communities Cand scient thesis 2002 Department of Biology, University of Oslo 59

[B51] Clemmensen KE Michelsen A Integrated long-term responses of an arctic-alpine willow and associated ectomycorrhizal fungi to an altered environment Canadian Journal of Botany-Revue Canadienne De Botanique 2006 84 5 831 843 10.1139/B06-039

[B52] Carlsen TA Molecular diversity of root endophytes in an alpine *Bistorta vivipara*-*Kobresia myosuroides *plant community Cand scient thesis 2002 Department of Biology, University of Oslo 53

[B53] Harrington TJ Mitchell DT Characterization of *Dryas octopetala *ectomycorrhizas from limestone karst vegetation, western Ireland Canadian Journal of Botany-Revue Canadienne De Botanique 2002 80 9 970 982 10.1139/b02-082

[B54] Harrington TJ Mitchell DT Ectomycorrhizas associated with a relict population of *Dryas octopetala *in the Burren, western Ireland II. Composition, structure and temporal variation in the ectomycorrhizal community Mycorrhiza 2005 15 6 435 445 10.1007/s00572-005-0348-3 15726434

[B55] Harrington TJ Mitchell DT Ectomycorrhizas associated with a relict population of *Dryas octopetala *in the Burren, western Ireland. I. Distribution of ectomycorrhizas in relation to vegetation and soil characteristics Mycorrhiza 2005 15 6 425 433 10.1007/s00572-005-0347-4 15726435

[B56] Lilleskov EA Fahey TJ Horton TR Lovett GM Belowground ectomycorrhizal fungal community change over a nitrogen deposition gradient in Alaska Ecology 2002 83 1 104 115 10.1890/0012-9658(2002)083[0104:BEFCCO]2.0.CO;2

[B57] Mehmann B Egli S Braus GH Brunner I Coincidence between molecularly or morphologically classified ectomycorrhizal morphotypes and fruitbodies in a spruce forest Biotechnology of Ectomycorrhizae 1995 41 52

[B58] Taylor AFS Fungal diversity in ectomycorrhizal communities: sampling effort and species detection Plant and Soil 2002 244 1-2 19 28 10.1023/A:1020279815472

[B59] Vrålstad T Schumacher T Taylor AFS Mycorrhizal synthesis between fungal strains of the *Hymenoscyphus ericae *aggregate and potential ectomycorrhizal and ericoid hosts New Phytologist 2002 153 1 143 152 10.1046/j.0028-646X.2001.00290.x

[B60] Haug I Agerer R Hebeloma velutipes Colour atlas of ectomycorrhizae 2002 Einhorn-Verlag: Schwäbisch Gmünd, Germany, plate 150

[B61] Beenken L Agerer R Inocybe terrigena Colour atlas of ectomycorrhizae 1996 Einhorn-Verlag: Schwäbisch Gmünd, Germany, plate 97

[B62] Agerer R Agerer R Tomentella albomarginata Colour atlas of ectomycorrhizae 1996 Einhorn-Verlag: Schwäbisch Gmünd, Germany, plate 111

[B63] Gronbach E and Agerer R Agerer R Cortinarius obtusus Colour atlas of ectomycorrhizae 1988 Einhorn-Verlag: Schwäbisch Gmünd, Germany, plate 12

[B64] Kõljalg U Dahlberg A Taylor AFS Larsson E Hallenberg N Stenlid J Larsson KH Fransson PM Karen O Jonsson L Diversity and abundance of resupinate thelephoroid fungi as ectomycorrhizal symbionts in Swedish boreal forests Molecular Ecology 2000 9 12 1985 1996 10.1046/j.1365-294X.2000.01105.x 11123611

[B65] Robinson CH Cold adaptation in Arctic and Antarctic fungi New Phytologist 2001 151 2 341 353 10.1046/j.1469-8137.2001.00177.x

[B66] Haselwandter K Read DJ The significance of a root-fungus association in 2 *Carex *species of high-alpine plant communities Oecologia 1982 53 3 352 354 10.1007/BF00389012 28311739

[B67] Newsham KK Upson R Read DJ Mycorrhiza and dark septate root endophytes in polar regions Fungal Ecology 2009 2 10 20 10.1016/j.funeco.2008.10.005 19495811

[B68] Ruotsalainen AL Tuomi J Vare H A model for optimal mycorrhizal colonization along altitudinal gradients Silva Fennica 2002 36 3 681 694

[B69] Jumpponen A Dark septate endophytes - are they mycorrhizal? Mycorrhiza 2001 11 4 207 211 10.1007/s005720100112

[B70] Grünig CR Queloz V Sieber TN Holdenrieder O Dark septate endophytes (DSE) of the *Phialocephala fortinii *s.l. - *Acephala applanata *species complex in tree roots: classification, population biology, and ecology Botany-Botanique 2008 86 12 1355 1369 10.1139/B08-108

[B71] Menkis A Vasiliauskas R Taylor AFS Stenlid J Finlay R Fungal communities in mycorrhizal roots of conifer seedlings in forest nurseries under different cultivation systems, assessed by morphotyping, direct sequencing and mycelial isolation Mycorrhiza 2005 16 1 33 41 10.1007/s00572-005-0011-z 16177926

[B72] Grünig CR Sieber TN Rogers SO Holdenrieder O Spatial distribution of dark septate endophytes in a confined forest plot Mycological Research 2002 106 832 840 10.1017/S0953756202005968

[B73] Addy HD Hambleton S Currah RS Distribution and molecular characterization of the root endophyte *Phialocephala fortinii *along an environmental gradient in the boreal forest of Alberta Mycological Research 2000 104 1213 1221 10.1017/S0953756200002896

[B74] Vrålstad T Myhre E Schumacher T Molecular diversity and phylogenetic affinities of symbiotic root-associated ascomycetes of the Helotiales in burnt and metal polluted habitats New Phytologist 2002 155 1 131 148 10.1046/j.1469-8137.2002.00444.x 33873290

[B75] Tedersoo L Koljalg U Hallenberg N Larsson KH Fine scale distribution of ectomycorrhizal fungi and roots across substrate layers including coarse woody debris in a mixed forest New Phytologist 2003 159 1 153 165 10.1046/j.1469-8137.2003.00792.x 33873690

[B76] Izzo A Agbowo J Bruns TD Detection of plot-level changes in ectomycorrhizal communities across years in an old-growth mixed-conifer forest New Phytologist 2005 166 2 619 630 10.1111/j.1469-8137.2005.01354.x 15819924

[B77] Anderson IC Cairney JWG Ectomycorrhizal fungi: exploring the mycelial frontier Fems Microbiology Reviews 2007 31 4 388 406 10.1111/j.1574-6976.2007.00073.x 17466031

[B78] Smith ME Douhan GW Rizzo DM Ectomycorrhizal community structure in a xeric Quercus woodland based on rDNA sequence analysis of sporocarps and pooled roots New Phytologist 2007 174 4 847 863 10.1111/j.1469-8137.2007.02040.x 17504467

[B79] von Humboldt A Cotta JG Ansichten der Natur mit wissenchaftlichen Erlauterungen 1808 Tübingen, Germany

[B80] Roy K Jablonski D Valentine JW Rosenberg G Marine latitudinal diversity gradients: Tests of causal hypotheses Proceedings of the National Academy of Sciences of the United States of America 1998 95 7 3699 3702 10.1073/pnas.95.7.3699 9520429 PMC19899

[B81] Price T Speciation in birds Speciation in birds 2008 Greenwood Village, Colorado, USA: Roberts and Company Publishers 1 470

[B82] Rahbek C Graves GR Multiscale assessment of patterns of avian species richness Proceedings of the National Academy of Sciences of the United States of America 2001 98 8 4534 4539 10.1073/pnas.071034898 11296292 PMC31869

[B83] Kaufman DM Willig MR Latitudinal patterns of mammalian species richness in the New World: the effects of sampling method and faunal group Journal of Biogeography 1998 25 4 795 805 10.1046/j.1365-2699.1998.2540795.x

[B84] Lyons SK Willig MR Species richness, latitude, and scale-sensitivity Ecology 2002 83 1 47 58 10.1890/0012-9658(2002)083[0047:SRLASS]2.0.CO;2

[B85] Gaston KJ Blackburn TM Spicer JI Rapoport's rule: time for an epitaph? Trends in Ecology & Evolution 1998 13 2 70 74 10.1016/s0169-5347(97)01236-6 21238203

[B86] Davies TJ Barraclough TG Savolainen V Chase MW Environmental causes for plant biodiversity gradients Philosophical Transactions of the Royal Society of London Series B-Biological Sciences 2004 359 1450 1645 1656 10.1098/rstb.2004.1524 PMC1693439 15519979

[B87] Arnold AE Lutzoni F Diversity and host range of foliar fungal endophytes: Are tropical leaves biodiversity hotspots? Ecology 2007 88 3 541 549 10.1890/05-1459 17503580

[B88] Neufeld JD Mohn WW Unexpectedly high bacterial diversity in arctic tundra relative to boreal forest soils, revealed by serial analysis of ribosomal sequence tags Applied and Environmental Microbiology 2005 71 10 5710 5718 10.1128/AEM.71.10.5710-5718.2005 16204479 PMC1266009

[B89] Tedersoo L Nara K General latitudinal gradient of biodiversity is reversed in ectomycorrhizal fungi New Phytologist 2010 185 2 351 354 10.1111/j.1469-8137.2009.03134.x 20088976

[B90] Allen MF Swenson W Querejeta JI Egerton-Warburton LM Treseder KK Ecology of mycorrhizae: A conceptual framework for complex interactions among plants and fungi Annual Review of Phytopathology 2003 41 271 303 10.1146/annurev.phyto.41.052002.095518 12730396

