# Supplementary material for: High diversity of root associated fungi in both alpine and arctic Dryas octopetala
Source: BMC Plant Biol. 2010 Nov 11;10:244. doi: 10.1186/1471-2229-10-244 (PMC3095326; doi:10.1186/1471-2229-10-244)
Supplement: Additional file 1 — List of basidiocarps used as reference sequences. [file 1471-2229-10-244-S1.DOC]

Additional file 1. List of basidiocarps used as reference sequences.

| Herbarium accession number | Taxon | Genbank accession no. |
| --- | --- | --- |
| 1_S1F14_a | *Russula* cf. *delica* | HQ445587 |
| 2_S1F6_a | *Cortinarius* cf. *albonigrellus* | HQ445588 |
| 3_S1F18_a | *Hebeloma* cf. *kuehneri* | HQ445589 |
| 4_S1F33_a | *Hebeloma* cf. *alpinum* | HQ445590 |
| 5_S1F33_b | *Cortinarius* cf. *minutulus* | HQ445591 |
| 6_S2F10_a | *Cortinarius* cf. *obtusus* | HQ445592 |
| 7_S2F10_b | *Cortinarius* cf. *obtusus* | HQ445593 |
| 8_S2F1_a | *Cortinarius* cf. *alpinus* | HQ445594 |
| 9_S2F16 | *Cortinarius* cf. *obtusus* | HQ445595 |
| 10_N1F4_1 | *Cortinarius* cf. *polaris* | HQ445596 |
| 11_N1F4_2 | *Cortinarius* cf. *alpinus* | HQ445597 |
| 14_N1F18_1 | *Cortinarius* cf. *alpinus* | HQ445598 |
| 15_N1F20_1 | *Cortinarius* cf. *alpinus* | HQ445599 |
| 16_N1F23_1 | *Cortinarius* cf. *alpinus* | HQ445600 |
| 17_N1F25_1 | *Cortinarius* cf. *polaris* | HQ445601 |
| 18_N1F26_1 | *Inocybe* cf. *fastigiata* | HQ445602 |
| 19_N1F29_F35 | *Omphalina* cf. *rivulicola* | HQ445603 |
| 20_N1F30_1 | *Galerina* cf. *pseudomycenopsis* | HQ445604 |
| 21_N1F31_1a | *Cortinarius* cf. *alpinus* | HQ445605 |
| 22_N1F31_1b | *Cortinarius* cf. *polaris* | HQ445606 |
| 23_N1F31_2 | *Entoloma* cf. *alpicolum* | HQ445607 |
| 24_N1F34_1 | *Cortinarius* cf. *alpinus* | HQ445608 |
| 25_N1F36_1 | *Cortinarius* cf. *polaris* | HQ445609 |
| 26_N2F2_1 | *Inocybe* cf. *fastigiata* | HQ445610 |
| 29_N3F13 | *Cortinarius* cf. *polaris* | HQ445611 |
| 30_N3F18 | *Hebeloma* cf. *alpinum* | HQ445612 |
| 31_N3F20 | *Inocybe* cf. *lacera* | HQ445613 |
| 32_N3F21 | *Mycena* cf. *galopus* | HQ445614 |
| 33_N3F23 | *Inocybe* cf. *flocculosa* | HQ445615 |
| 34_N3F25 | *Marasmius* cf. *epidryas* | HQ445616 |
| 35_N3F27 | *Omphalina* cf. *rivulicola* | HQ445617 |
| 36_N343 | *Cortinarius* cf. *alpinus* | HQ445618 |
| GG165_88 | *Clitocybe favrei* | GU234009 |
| O73608 | *Lactarius lanceolatus* | GU234010 |
| O71622 | *Russula maculata* | GU234011 |
| GG401_86 | *Cortinarius inops* | GU234012 |
| O50591 | *Cortinarius subtorvus* | GU234013 |
| O73061 | *Agaricus aristocratus* | GU234014 |
| GG23_88 | *Clitocybe festiva* | GU234015 |
| GG121_88 | *Collybia alkalivirens* | GU234016 |
| KH8 | *Russula delica* | GU234017 |
| O75453 | *Thelphora caryophylla* | GU234018 |
| O73576 | *Lactarius nanus* | GU234019 |
| GG212_86 | *Cortinarius minutulus* | GU234020 |
| GG181_88 | *Clitocybe festiva* | GU234021 |
| GG122_88 | *Collybia alkalivirens* | GU234022 |
| KH1 | *Russula nana* | GU234023 |
| O73614 | *Russula* sp. | GU234024 |
| O73575 | *Russula norvegica* | GU234025 |
| GG156_86 | *Cortinarius minutulus* | GU234026 |
| O73706 | *Lactarius glyciosmus* | GU234027 |
| GG185_88 | *Cystoderma arcticum* | GU234028 |
| KH10 | *Cortinarius* cfr. *inconspicuus* | GU234029 |
| O73612 | *Russula* sp. | GU234030 |
| O73652 | *Russula saliceticola* | GU234031 |
| GG213_86 | *Cortinarius rufostriatus* | GU234032 |
| O73053 | *Arrhenia lobata* | GU234033 |
| GG85_88 | *Coprinus martinii* | GU234034 |
| KH3 | *Russula norvegica* | GU234035 |
| O73645 | *Cortinarius alpinus* | GU234036 |
| GG303_86 | *Cortinarius* sp. | GU234037 |
| GG228_86 | *Cortinarius phaechrous* | GU234038 |
| O73153 | *Russsula xerampelina* | GU234039 |
| O73055 | *Cortinarius absarokensis* | GU234040 |
| GG218_88 | *Clitocybe langei* | GU234041 |
| KH4 | *Russula delica* | GU234042 |
| KH12 | *Cortinarius obtusus* | GU234043 |
| GG91_86 | *Cortinarius phaeopygmaeus* | GU234044 |
| O50573 | *Cortinarius anomalus* | GU234045 |
| GG290_86 | *Lactarius dryadophilus* | GU234046 |
| O73136 | *Russula violaceoincarnata* | GU234047 |
| GG31_88 | *Arrhenia acerosa* | GU234048 |
| GG219_88 | *Clitocybe langei* | GU234049 |
| GG160_88 | *Galerina miniophila* | GU234050 |
| KH5 | *Lactarius pseudovidus* | GU234051 |
| KH13 | *Cortinarius obtusus* | GU234052 |
| O73613 | *Russula* sp. | GU234053 |
| O73634 | *Russula chamiteae* | GU234054 |
| GG344_86 | *Cortinarius pauperculus* | GU234055 |
| O50564 | *Cortinarius delibutus* | GU234056 |
| GG124_88 | *Galerina pseudomycenopsis* | GU234057 |
| KH14 | *Cortinarius subtorvus* | GU234058 |
| O71620 | *Lactarius violascens* | GU234059 |
| GG346_86 | *Cortinarius tenebricus* | GU234060 |
| GG269_86 | *Cortinarius pauperculus* | GU234061 |
| GG415_86 | *Lactarius mammosus* | GU234062 |
| O73052 | *Russula chamiteae* | GU234063 |
| GG51_88 | *Clitocybe dryadicola* | GU234064 |
| GG173_88 | *Clitocybe paxillus* | GU234065 |
| GG239_86 | *Melanoleuca cognata* | GU234066 |
| GG215_88 | *Hypholoma elongatipes* | GU234067 |
| KH15 | *Cortinarius* cfr. sp. | GU234068 |
| KH23 | *Cortinarius scotoides* | GU234069 |
| KH30 | *Cortinarius alpinus* | GU234070 |
| KH38 | *Inocybe dulcamara* | GU234071 |
| KH46 | *Inocybe fastigiata* | GU234072 |
| KH54 | *Entoloma alpicola* | GU234073 |
| KH62 | *Galerina pseudomycenopsis* | GU234074 |
| KH70 | *Lycoperdon lividum* | GU234075 |
| KH78 | *Fayodia* sp. | GU234076 |
| GG281_81 | *Melanoleuca cognata* | GU234077 |
| GG211_88 | *Hypholoma myosotis* | GU234078 |
| KH16 | *Cortinarius* sp. | GU234079 |
| KH23B | *Cortinarius* cfr.  *scotoides* | GU234080 |
| KH31 | *Cystoderma arcticum* | GU234081 |
| KH39 | *Inocybe dulcamara* | GU234082 |
| KH47 | *Hebeloma mesophaeum* | GU234083 |
| KH71 | *Clitocybe* cfr.  *festiva* | GU234084 |
| GG253_86 | *Callocybe onchina* | GU234085 |
| KH17 | *Cortinarius* cfr. *helobius* | GU234086 |
| KH24 | *Cortinarius alpinus* | GU234087 |
| KH32 | *Lactarius nanus* | GU234088 |
| KH40 | *Inocybe calamistrata* | GU234089 |
| KH48 | *Hebeloma marginatulum* | GU234090 |
| KH56 | *Entoloma alpicola* | GU234091 |
| KH64 | *Hebeloma* cfr. *minus* | GU234092 |
| KH72 | *Omphalina rustica* | GU234093 |
| GG255_86 | *Calocybe onchina* | GU234094 |
| GG438_86 | *Mycena atroalboides* | GU234095 |
| KH25 | *Cortinarius alpinus* | GU234096 |
| KH49 | *Hebeloma* cfr. *kuehneri* | GU234097 |
| KH57 | *Laccaria laccata* | GU234098 |
| KH65 | *Hebeloma alpinum* | GU234099 |
| KH73 | *Omphalina grisella* | GU234100 |
| O50495 | *Clitocybe dryadicola* | GU234101 |
| GG172_88 | *Lyophyllum connatum* | GU234102 |
| KH19 | *Cortinarius* cfr. *albonigrellus* | GU234103 |
| KH34 | *Inocybe boltonii* | GU234104 |
| KH42 | *Inocybe lacera* | GU234105 |
| KH50 | *Hebeloma* cfr. *kuehneri* | GU234106 |
| KH58 | *Marasmius epidryas* | GU234107 |
| KH66 | *Hebeloma alpinum* | GU234108 |
| KH74 | *Omphalina rivulicola* | GU234109 |
| O50514 | *Clitocybe paxillus* | GU234110 |
| GG188_86 | *Mycena citrinomarginata* | GU234111 |
| GG435_86 | *Mycena olivaceomarginata* | GU234112 |
| KH20 | *Cortinarius* cfr. *albonigrellus* | GU234113 |
| KH35 | *Inocybe boltonii* | GU234114 |
| KH59 | *Marasmius epidryas* | GU234115 |
| KH67 | *Hebeloma alpinum* | GU234116 |
| KH75 | *Omphalina rivulicola* | GU234117 |
| GG432_86 | *Mycena filopes* | GU234118 |
| GG436_86 | *Mycena olivaceomarginata* | GU234119 |
| KH28 | *Russula norvegica* | GU234120 |
| KH44 | *Inocybe* cfr. *lacera* | GU234121 |
| KH52 | *Inocybe* cfr. *hirtella* | GU234122 |
| KH60 | *Galerina* cfr. *arctica* | GU234123 |
| KH68 | *Hebeloma* cfr. *bruchetii* | GU234124 |
| KH76 | *Lycoperdon* sp. | GU234125 |
| O50524 | *Coprinus martinii* | GU234126 |
| GG249_86 | *Mycena pura* | GU234127 |
| KH29 | *Cortinarius polaris* | GU234128 |
| KH37 | *Inocybe dulcamara* | GU234129 |
| KH45 | *Inocybe fastigiata* | GU234130 |
| KH53 | *Inocybe* cfr. *hirtella* | GU234131 |
| KH61 | *Galerina pseudomycenopsis* | GU234132 |
| KH69 | *Hebeloma* sp. | GU234133 |
| KH77 | *Mycena* cfr. *galopus* | GU234134 |
| GG186_86 | *Naucoria (syn. Alnicola) tantilla* | GU234135 |
| GG119_88 | *Rickenella setipes* | GU234136 |
| O73586 | *Lyophyllum* sp. | GU234137 |
| O73639 | *Mycena simia* | GU234138 |
| GG146_86 | *Naucoria (syn. Alnicola) tantilla* | GU234139 |
| GG102_81 | *Psilocybe magnivelloris* | GU234140 |
| O73581 | *Collybia alkalivirens* | GU234141 |
| O73604 | *Fayodia leucophylla* | GU234142 |
| O73615 | *Hypholoma myosotis* | GU234143 |
| GG106_88 | *Omphalina chionophila* | GU234144 |
| GG312_86 | *Hygrocybe citrinopallida* | GU234145 |
| O73656 | *Mycena cinerella* | GU234146 |
| O73621 | *Lactarius uvidus* | GU234147 |
| O73640 | *Clavaria falcata* | GU234148 |
| GG355_86 | *Hydropus scabripes* | GU234149 |
| O73653 | *Mycena citrinomarginata* | GU234150 |
| O73633 | *Cystoderma* sp. | GU234151 |
| GG104_88 | *Pholiota* sp. | GU234152 |
| O73607 | *Coprinus* sp. | GU234153 |
| GG297_81 | *Cystoderma arcticum* | GU234154 |
| O73610 | *Leccinum rotundifoliae* | GU234155 |
| O73579 | *Inocybe* sp. | GU234156 |
| GG126_86 | *Laccaria pumila* | GU234157 |
| GG238_86 | *Lactarius obscuratus* | GU234158 |
| GG109_86 | *Lactarius obscuratus* | GU234159 |
| O73643 | *Hebeloma* sp. | GU234160 |
| GG125_86 | *Laccaria pumila* | GU234161 |
| GG437_86 | *Mycena atroalboides* | GU234162 |
| GG154_88 | *Rickenella fibula* | GU234163 |
| GG105_88 | *Pholiota* sp. | GU234164 |
| O73637 | *Mycena pura* | GU234165 |
