# Supplementary material for: High diversity of root associated fungi in both alpine and arctic Dryas octopetala
Source: BMC Plant Biol. 2010 Nov 11;10:244. doi: 10.1186/1471-2229-10-244 (PMC3095326; doi:10.1186/1471-2229-10-244)
Supplement: Additional file 2 — List of all unique sequences with their best matches in GenBank/UNITE/basidiocarp reference sequences. [file 1471-2229-10-244-S2.DOC]

**Additional file 2 - list of all unique sequences with their best matches in GenBank/UNITE/basidiocarp reference sequences.**

^1^*Dryas octopetala* plant root system sampled. First letter and number indicate sub-locality number, second letter and number indicates *Dryas* sampled from square number x in the 6 x 6 m grid (E.g., F1D13; Finse, sub-locality number 1, *Dryas* sampled from square number 13). ^2^Sequence number. ^3^The number of clones detected for the respective ITS genotype. ^4^Number of genotypes observed within plant root system. ^5^Number of genotypes observed among plant root systems. ^6^Operational taxonomic unit number. ^7^Affiliation of ITS sequence to fungal order, letters inside brackets indicates Basidiomycota (B), Ascomycota (A), Zygomycota (Z), and Glomeromycota (G). ^8^Accession number of sequences with best match to environmental sequences in GenBank/UNITE/Herbarium/sampled sporocarp. ^9^Taxonomic affiliation, H inside bracket indicates hyphomycete. ^10^Match score of query sequence with reference sequence in GenBank/UNITE/local database. ^11^Query coverage with reference sequence. ^12^Maximum identity with reference sequence. ^13^Accession number of the unique environmental sequences accessioned in GenBank.

| **Plant^1^** | **Sequence^2^** | **#3** | **#GT/P^4^** | **GT AP^5^** | **OTU#^6^** | **Order^7^** | **Accession #^8^** | **Identification^9^** | **Score^10^** | **QC^11^** | **Ident.^12^** | **Acc.# E.S.^13^** |
| --- | --- | --- | --- | --- | --- | --- | --- | --- | --- | --- | --- | --- |
| F1D13 | F1D13_21 | 7 | 1 | 1 | 1 | Cantharellales (B) | DQ974710.1 | *Clavulina* cf. *cristata* | 569 | 98 % | 84 % | HQ444981 |
|  | F1D13_24 | 1 | 2 | 2 | 2 | Agaricales (B) | 30_N3F18 | *Hebeloma alpinum* | 1150 | 100 % | 99 % | HQ444984 |
|  | F1D13_1 | 3 | 3 | 3 | 2 | Agaricales (B) | 30_N3F18 | *Hebeloma alpinum* | 1205 | 100 % | 98 % | HQ444963 |
|  | F1D13_19 | 1 | 4 | 4 | 3 | Helotiales (A) | AY347412.1 | *Phialocephala helvetica* | 828 | 100 % | 97 % | HQ444979 |
|  | F1D13_23 | 1 | 5 | 5 | 4 | Helotiales (A) | AY880935.1 | *Phialocephala fortinii* | 878 | 100 % | 99 % | HQ444983 |
|  | F1D13_16 | 4 | 6 | 6 | 5 | Pezizales (A) | DQ200835.1 | *Trichophaea woolhopeia* | 737 | 86 % | 95 % | HQ444976 |
|  | F1D13_20 | 1 | 7 | 7 | 6 | Dothideomycetes (A) | AJ510274.1 | *Cenococcum geophilum* | 813 | 100 % | 99 % | HQ444980 |
|  | F1D13_5 | 1 | 8 | 8 | 7 | Pezizomycotina (A) | AY729938.1 | *Lunulospora curvula* | 813 | 100 % | 97 % | HQ444966 |
|  | F1D13_14 | 1 | 9 | 9 | 8 | Agaricales (B) | UDB002161 | *Cortinarius diasemospermus* | 924 | 100 % | 98 % | HQ444974 |
|  | F1D13_8 | 1 | 10 | 10 | 9 | Agaricales (B) | AM882714.1 | *Inocybe egenula* | 915 | 98 % | 96 % | HQ444968 |
|  | F1D13_11 | 1 | 11 | 11 | 10 | Thelephorales (B) | EU819523.1 | *Tomentella stuposa* | 933 | 99 % | 95 % | HQ444971 |
|  | F1D13_17 | 1 | 12 | 12 | 11 | Cantharellales (B) | UDB002253 | *Sistotrema alboluteum* | 295 | 29 % | 96 % | HQ444977 |
|  | F1D13_25 | 1 | 13 | 13 | 12 | Helotiales (A) | AJ430219.1 | *Lachnum pygmaeum* | 848 | 100 % | 98 % | HQ444985 |
| F1D20 | F1D20_23 | 22 | 1 | 14 | 13 | Sebacinales (B) | AJ966754.1 | *Sebacina epigaea* | 688 | 98 % | 90 % | HQ445009 |
|  | F1D20_9 | 1 | 2 | 15 | 7 | Ascomycota (A) | AY729938.1 | *Lunulospora curvula* | 802 | 100 % | 97 % | HQ444995 |
|  | F1D20_20 | 1 | 3 | 16 | 14 | Leotiomycetes (A) | AY706329.1 | *Leohumicola minima* | 625 | 100 % | 90 % | HQ445006 |
| F2D1 | F2D1_13 | 1 | 1 | 17 | 15 | Sebacinales (B) | EF655701.1 | *Sebacina incrustans* | 979 | 99 % | 100 % | HQ445020 |
|  | F2D1_17 | 1 | 2 | 18 | 15 | Sebacinales (B) | EF655701.1 | *Sebacina incrustans* | 963 | 99 % | 99 % | HQ445022 |
|  | F2D1_30 | 1 | 3 | 19 | 6 | Dothideomycetes (A) | AY940649.1 | *Cenococcum geophilum* | 832 | 100 % | 99 % | HQ445034 |
|  | F2D1_1 | 2 | 4 | 20 | 6 | Dothideomycetes (A) | AF495462.1 | *Cenococcum geophilum* | 809 | 100 % | 98 % | HQ445011 |
|  | F2D1_5 | 1 | 5 | 21 | 16 | Cantharellales (B) | EU819415.1 | *Clavulina cristata* | 889 | 99 % | 92 % | HQ445013 |
|  | F2D1_6 | 1 | 6 | 22 | 16 | Cantharellales (B) | EU819415.1 | *Clavulina cristata* | 926 | 99 % | 93 % | HQ445014 |
|  | F2D1_18 | 1 | 7 | 23 | 17 | Agaricales (B) | AM882912.1 | *Inocybe* cf. *nitidiuscula* | 1074 | 98 % | 99 % | HQ445023 |
|  | F2D1_31 | 3 | 8 | 24 | 17 | Agaricales (B) | AM882912.1 | *Inocybe* cf. *nitidiuscula* | 1081 | 98 % | 99 % | HQ445033 |
|  | F2D1_9 | 3 | 9 | 25 | 17 | Agaricales (B) | AM882912.1 | *Inocybe* cf. *nitidiuscula* | 1081 | 98 % | 99 % | HQ445017 |
|  | F2D1_20 | 1 | 10 | 26 | 17 | Agaricales (B) | AM882912.1 | *Inocybe* cf. *nitidiuscula* | 1064 | 98 % | 98 % | HQ445025 |
|  | F2D1_28 | 1 | 11 | 27 | 17 | Agaricales (B) | AM882912.1 | *Inocybe* cf. *nitidiuscula* | 1064 | 98 % | 98 % | HQ445031 |
|  | F2D1_27 | 2 | 12 | 28 | 18 | Agaricales (B) | AM882931.1 | *Inocybe subporospora* | 623 | 96 % | 86 % | HQ445030 |
|  | F2D1_21 | 1 | 13 | 29 | 18 | Agaricales (B) | AM882931.1 | *Inocybe subporospora* | 628 | 96 % | 86 % | HQ445026 |
|  | F2D1_25 | 1 | 14 | 30 | 18 | Agaricales (B) | AM882931.1 | *Inocybe subporospora* | 617 | 96 % | 86 % | HQ445029 |
|  | F2D1_7 | 1 | 15 | 31 | 19 | Thelephorales (B) | AJ889981.1 | *Tomentella bryophila* | 876 | 100 % | 93 % | HQ445015 |
|  | F2D1_11 | 1 | 16 | 32 | 20 | Ascomycota (A) | AY729937.1 | *Gyoerffyella rotula* | 732 | 100 % | 94 % | HQ445019 |
|  | F2D1_29 | 1 | 17 | 33 | 8 | Agaricales (B) | KH10 | *Cortinarius inconspicuus* | 993 | 100 % | 99 % | HQ445032 |
|  | F2D1_23 | 1 | 18 | 34 | 21 | Hypocreales (A) | EF488439.1 | *Cordyceps sinensis* | 684 | 98 % | 92 % | HQ445028 |
| F2D35 | F2D35_2 | 8 | 2 | 36 | 23 | Russulales (B) | DQ422015.1 | *Russula* cf. *maculata* | 828 | 100 % | 90 % | HQ445036 |
|  | F2D35_17 | 2 | 3 | 37 | 23 | Russulales (B) | DQ422015.1 | *Russula* cf. *maculata* | 822 | 100 % | 90 % | HQ445046 |
|  | F2D35_31 | 3 | 4 | 38 | 24 | Thelephorales (B) | AJ889981.1 | *Tomentella bryophila* | 905 | 100 % | 94 % | HQ445057 |
|  | F2D35_10 | 2 | 5 | 39 | 25 | Agaricales (B) | UDB001031 | *Cortinarius decipiens* | 989 | 100 % | 99 % | HQ445042 |
|  | F2D35_11 | 3 | 6 | 40 | 25 | Agaricales (B) | UDB001031 | *Cortinarius decipiens* | 971 | 100 % | 99 % | HQ445043 |
|  | F2D35_4 | 1 | 7 | 41 | 6 | Dothideomycetes (A) | AY940649.1 | *Cenococcum geophilum* | 837 | 100 % | 100 % | HQ445038 |
|  | F2D35_19 | 1 | 8 | 42 | 26 | Leotiomycetes (A) | AY706329.1 | *Leohumicola minima* | 798 | 100 % | 97 % | HQ445048 |
|  | F2D35_5 | 1 | 9 | 43 | 27 | Teloschistales (A) | EU639615.1 | *Teloschistes lacunosus* | 316 | 45 % | 95 % | HQ445039 |
|  | F2D35_20 | 3 | 10 | 35 | 22 | Thelephorales (B) | AJ889981.1 | *Tomentella bryophila* | 819 | 100 % | 91 % | HQ445049 |
| F3D13 | F3D13_23 | 14 | 1 | 41 | 6 | Dothideomycetes (A) | AY940649.1 | *Cenococcum geophilum* | 818 | 100 % | 100 % | HQ445080 |
|  | F3D13_4 | 1 | 2 | 44 | 6 | Dothideomycetes (A) | AJ510274.1 | *Cenococcum geophilum* | 801 | 100 % | 99 % | HQ445062 |
|  | F3D13_22 | 1 | 3 | 45 | 6 | Dothideomycetes (A) | AJ510274.1 | *Cenococcum geophilum* | 798 | 100 % | 99 % | HQ445079 |
|  | F3D13_17 | 2 | 4 | 46 | 28 | Helotiales (A) | DQ485204.1 | *Cadophora finlandica* | 820 | 100 % | 98 % | HQ445074 |
|  | F3D13_2 | 1 | 5 | 47 | 29 | Botryosphaeriales (A) | FJ037758.1 | *Botryosphaeria australis* | 376 | 62 % | 85 % | HQ445060 |
|  | F3D13_5 | 2 | 6 | 48 | 30 | Thelephorales (B) | DQ974776.1 | *Tomentella fuscocinerea* | 949 | 100 % | 95 % | HQ445063 |
|  | F3D13_9 | 1 | 7 | 49 | 4 | Helotiales (A) | AY078130.1 | Cf. *Phialocephala fortinii* | 850 | 100 % | 98 % | HQ445067 |
|  | F3D13_20 | 1 | 8 | 50 | 4 | Helotiales (A) | AY078130.1 | Cf. *Phialocephala fortinii* | 854 | 100 % | 98 % | HQ445077 |
|  | F3D13_8 | 1 | 9 | 51 | 22 | Thelephorales (B) | AJ889981.1 | *Tomentella bryophila* | 834 | 100 % | 91 % | HQ445066 |
| F3D34 | F3D34_7 | 6 | 1 | 52 | 8 | Agaricales (B) | UDB001230 | *Cortinarius diasemospermus* | 1017 | 100 % | 100 % | HQ445089 |
|  | F3D34_26 | 12 | 2 | 53 | 4 | Helotiales (A) | FJ031032.1 | *Phialocephala fortinii* | 868 | 100 % | 99 % | HQ445105 |
|  | F3D34_27 | 1 | 3 | 54 | 31 | Thelephorales (B) | AJ889981.1 | *Tomentella bryophila* | 874 | 100 % | 93 % | HQ445106 |
|  | F3D34_14 | 1 | 4 | 55 | 6 | Dothideomycetes (A) | AJ510274.1 | *Cenococcum geophilum* | 807 | 100 % | 99 % | HQ445094 |
|  | F3D34_24 | 1 | 5 | 56 | 6 | Dothideomycetes (A) | AY940649.1 | *Cenococcum geophilum* | 812 | 100 % | 99 % | HQ445104 |
|  | F3D34_1 | 1 | 6 | 57 | 32 | Dothideomycetes (A) | AY129290.1 | *Pseudeurotium ovale var.milkoi* | 412 | 94 % | 81 % | HQ445083 |
|  | F3D34_2 | 1 | 7 | 58 | 33 | Agaricales (B) | AM882931.1 | *Inocybe subporospora* | 583 | 97 % | 82 % | HQ445084 |
|  | F3D34_3 | 1 | 8 | 59 | 34 | Sebacinales (B) | AF384862.1 | *Tremellodendron pallidum* | 814 | 98 % | 93 % | HQ445085 |
| N1D12 | N1D12_16 | 2 | 1 | 33 | 8 | Agaricales (B) | KH10 | *Cortinarius inconspicuus* | 993 | 99 % | 100 % | HQ445168 |
|  | N1D12_4 | 8 | 2 | 65 | 8 | Agaricales (B) | KH10 | *Cortinarius inconspicuus* | 1001 | 99 % | 100 % | HQ445157 |
|  | N1D12_5 | 1 | 3 | 66 | 22 | Thelephorales (B) | AJ889981.1 | *Tomentella bryophila* | 843 | 100 % | 92 % | HQ445158 |
|  | N1D12_3 | 2 | 4 | 67 | 22 | Thelephorales (B) | AJ889981.1 | *Tomentella bryophila* | 852 | 100 % | 92 % | HQ445156 |
|  | N1D12_30 | 2 | 5 | 68 | 36 | Sebacinales (B) | EU819519.1 | *Sebacina* aff. *epigaea* | 852 | 99 % | 95 % | HQ445177 |
|  | N1D12_6 | 2 | 6 | 69 | 2 | Agaricales (B) | 30_N3F18 | *Hebeloma alpinum* | 1166 | 98 % | 100 % | HQ445159 |
|  | N1D12_29 | 1 | 7 | 70 | 37 | Agaricales (B) | AF124682.1 | *Hebeloma sinapizans* | 985 | 98 % | 97 % | HQ445176 |
|  | N1D12_14 | 1 | 8 | 71 | 37 | Agaricales (B) | AF124682.1 | *Hebeloma sinapizans* | 998 | 98 % | 97 % | HQ445166 |
|  | N1D12_9 | 1 | 9 | 72 | 37 | Agaricales (B) | AF124682.1 | *Hebeloma sinapizans* | 1032 | 98 % | 97 % | HQ445162 |
|  | N1D12_26 | 1 | 10 | 73 | 38 | Hypocreales (A) | EF029217.1 | *Fusidium griseum* | 567 | 100 % | 86 % | HQ445173 |
|  | N1D12_22 | 1 | 11 | 74 | 39 | Thelephorales (B) | DQ974776.1 | *Tomentella fuscocinerea* | 910 | 100 % | 94 % | HQ445171 |
|  | N1D12_10 | 1 | 12 | 75 | 40 | Thelephorales (B) | DQ068972.1 | *Tomentella lilacinogrisea* | 875 | 100 % | 93 % | HQ445163 |
|  | N1D12_15 | 1 | 13 | 76 | 41 | Agaricales (B) | AF335452.1 | *Inocybe* cf. *flocculosa* | 1086 | 100 % | 98 % | HQ445167 |
| N1D35 | N1D35_12 | 2 | 1 | 77 | 42 | Cantharellales (B) | DQ974710.1 | *Clavulina* cf. *cristata* | 517 | 98 % | 79 % | HQ445185 |
|  | N1D35_3 | 3 | 2 | 78 | 42 | Cantharellales (B) | DQ974710.1 | *Clavulina* cf. *cristata* | 515 | 98 % | 79 % | HQ445180 |
|  | N1D35_7 | 3 | 3 | 79 | 43 | Thelephorales (B) | AY010275.1 | *Tomentella subclavigera* | 917 | 100 % | 95 % | HQ445183 |
|  | N1D35_14 | 9 | 4 | 80 | 43 | Thelephorales (B) | AY010275.1 | *Tomentella subclavigera* | 935 | 100 % | 95 % | HQ445187 |
|  | N1D35_30 | 1 | 5 | 81 | 44 | Thelephorales (B) | DQ974772.1 | *Tomentella atramentaria* | 924 | 100 % | 95 % | HQ445200 |
|  | N1D35_24 | 1 | 6 | 82 | 45 | Thelephorales (B) | AF272941.1 | *Tomentella lapidum* | 854 | 92 % | 94 % | HQ445196 |
|  | N1D35_23 | 1 | 7 | 83 | 46 | Thelephorales (B) | EU819523.1 | *Tomentella stuposa* | 861 | 99 % | 92 % | HQ445195 |
|  | N1D35_13 | 1 | 8 | 84 | 28 | Helotiales (A) | AF486119.1 | *Phialophora finlandia* | 809 | 100 % | 98 % | HQ445186 |
|  | N1D35_17 | 1 | 9 | 85 | 47 | Agaricales (B) | GG346_86 | *Cortinarius tenebricus* | 1011 | 99 % | 100 % | HQ445190 |
|  | N1D35_20 | 1 | 10 | 86 | 48 | Helotiales (A) | AY805585.1 | *Cadophora malorum* | 690 | 90 % | 95 % | HQ445193 |
|  | N1D35_29 | 1 | 11 | 87 | 49 | Hypocreales (A) | EF641861.1 | *Verticillium biguttatum* | 443 | 97 % | 81 % | HQ445199 |
| N2D5 | N2D5_5 | 1 | 1 | 88 | 50 | Thelephorales (B) | DQ974771.1 | *Thelephora anthocephala* | 897 | 100 % | 94 % | HQ445207 |
|  | N2D5_3 | 2 | 2 | 89 | 50 | Thelephorales (B) | DQ974771.1 | *Thelephora anthocephala* | 911 | 100 % | 94 % | HQ445205 |
|  | N2D5_4 | 4 | 3 | 90 | 51 | Agaricales (B) | AM882863.1 | *Inocybe dulcamara* | 1021 | 97 % | 95 % | HQ445206 |
|  | N2D5_13 | 1 | 4 | 91 | 52 | Thelephorales (B) | UDB000034 | *Tomentella subtestacea* | 870 | 94 % | 100 % | HQ445214 |
|  | N2D5_23 | 2 | 5 | 92 | 52 | Thelephorales (B) | UDB000034 | *Tomentella subtestacea* | 880 | 94 % | 100 % | HQ445224 |
|  | N2D5_17 | 3 | 6 | 93 | 40 | Thelephorales (B) | DQ068972.1 | *Tomentella lilacinogrisea* | 879 | 100 % | 93 % | HQ445218 |
|  | N2D5_16 | 2 | 7 | 94 | 33 | Agaricales (B) | AM882931.1 | *Inocybe subporospora* | 580 | 96 % | 82 % | HQ445217 |
|  | N2D5_12 | 5 | 8 | 95 | 33 | Agaricales (B) | AM882931.1 | *Inocybe subporospora* | 580 | 97 % | 82 % | HQ445213 |
|  | N2D5_20 | 1 | 9 | 96 | 53 | Leotiomycetes (A) | AY706325.1 | *Leohumicola verrucosa* | 630 | 100 % | 90 % | HQ445221 |
|  | N2D5_6 | 1 | 10 | 97 | 6 | Dothideomycetes (A) | AY534205.1 | *Cenococcum geophilum* | 805 | 100 % | 99 % | HQ445208 |
|  | N2D5_11 | 1 | 11 | 98 | 48 | Helotiales (A) | AY805585.1 | *Cadophora malorum* | 704 | 90 % | 95 % | HQ445212 |
|  | N2D5_24 | 1 | 12 | 99 | 8 | Agaricales (B) | KH10 | *Cortinarius inconspicuus* | 979 | 99 % | 100 % | HQ445225 |
| N2D9 | N2D9_28 | 4 | 1 | 100 | 54 | Cantharellales (B) | DQ974710.1 | *Clavulina* cf. *cristata* | 569 | 98 % | 82 % | HQ445250 |
|  | N2D9_4 | 4 | 2 | 101 | 55 | Thelephorales (B) | DQ974771.1 | *Thelephora anthocephala* | 913 | 100 % | 94 % | HQ445230 |
|  | N2D9_19 | 4 | 3 | 102 | 56 | Agaricales (B) | 26_N2F2_1 | *Inocybe fastigiata* | 1225 | 100 % | 100 % | HQ445243 |
|  | N2D9_18 | 1 | 4 | 58 | 33 | Agaricales (B) | AM882931.1 | *Inocybe subporospora* | 583 | 97 % | 82 % | HQ445242 |
|  | N2D9_11 | 3 | 5 | 94 | 33 | Agaricales (B) | AM882931.1 | *Inocybe subporospora* | 585 | 96 % | 82 % | HQ445236 |
|  | N2D9_3 | 1 | 6 | 103 | 2 | Agaricales (B) | 30_N3F18 | *Hebeloma alpinum* | 1197 | 100 % | 99 % | HQ445229 |
|  | N2D9_13 | 4 | 7 | 104 | 2 | Agaricales (B) | 30_N3F18 | *Hebeloma alpinum* | 1181 | 100 % | 98 % | HQ445238 |
|  | N2D9_1 | 1 | 8 | 105 | 57 | Leotiomycetes (A) | AY706325.1 | *Leohumicola verrucosa* | 724 | 100 % | 94 % | HQ445227 |
|  | N2D9_10 | 1 | 9 | 106 | 6 | Dothideomycetes (A) | AY534205.1 | *Cenococcum geophilum* | 801 | 100 % | 99 % | HQ445235 |
|  | N2D9_26 | 1 | 10 | 107 | 58 | Helotiales (A) | DQ202516.1 | *Varicosporium delicatum* | 677 | 78 % | 94 % | HQ445248 |
| N3D4 | N3D4_4 | 1 | 1 | 107 | 59 | Chaetothyriales (A) | AJ244275.1 | *Sarcinomyces petricola* | 785 | 100 % | 92 % | HQ445278 |
|  | N3D4_7 | 2 | 2 | 109 | 60 | Lecanorales (A) | AY756496.1 | *Psilolechia leprosa* | 491 | 88 % | 84 % | HQ445281 |
|  | N3D4_21 | 6 | 3 | 110 | 61 | Agaricales (B) | 17_N1F25_1 | *Cortinarius polaris* | 1201 | 100 % | 100 % | HQ445295 |
|  | N3D4_9 | 4 | 4 | 111 | 61 | Agaricales (B) | 29_N3F13 | *Cortinarius polaris* | 1189 | 99 % | 100 % | HQ445283 |
|  | N3D4_10 | 3 | 5 | 112 | 62 | Sebacinales (B) | AJ966754.1 | *Sebacina epigaea* | 782 | 98 % | 93 % | HQ445284 |
|  | N3D4_15 | 2 | 6 | 113 | 48 | Helotiales (A) | AY805585.1 | *Cadophora malorum* | 719 | 90 % | 96 % | HQ445289 |
|  | N3D4_17 | 2 | 7 | 114 | 63 | Helotiales (A) | AF169309.1 | *Hymenoscyphus monotropae* | 664 | 100 % | 91 % | HQ445291 |
|  | N3D4_18 | 1 | 8 | 115 | 64 | Helotiales (A) | AY348582.1 | *Hymenoscyphus* cf. *fructigenus* | 809 | 99 % | 98 % | HQ445292 |
|  | N3D4_11 | 1 | 9 | 116 | 28 | Helotiales (A) | AF486119.1 | *Phialophora finlandia* | 814 | 100 % | 98 % | HQ445285 |
|  | N3D4_8 | 1 | 10 | 117 | 27 | Teloschistales (A) | EU639615.1 | *Teloschistes lacunosus* | 313 | 45 % | 95 % | HQ445282 |
|  | N3D4_20 | 1 | 11 | 118 | 65 | Halosphaeriales (A) | AY150224.1 | *Phaeonectriella lignicola* | 170 | 25 % | 83 % | HQ445294 |
| N3D28 | N3D28_6 | 1 | 1 | 119 | 66 | Agaricales (B) | AY669673.1 | *Cortinarius rubricosus* | 877 | 100 % | 97 % | HQ445255 |
|  | N3D28_2 | 10 | 2 | 120 | 47 | Agaricales (B) | GG346_86 | *Cortinarius tenebricus* | 1027 | 100 % | 100 % | HQ445252 |
|  | N3D28_16 | 2 | 3 | 121 | 67 | Acarosporales (A) | DQ525522.1 | *Pleopsidium gobiense* | 300 | 51 % | 89 % | HQ445264 |
|  | N3D28_10 | 2 | 4 | 122 | 21 | Coniochaetales (A) | AY198390.1 | *Coniochaeta ligniaria* | 688 | 100 % | 91 % | HQ445258 |
|  | N3D28_7 | 2 | 5 | 74 | 39 | Thelephorales (B) | DQ974776.1 | *Tomentella fuscocinerea* | 910 | 100 % | 94 % | HQ445256 |
|  | N3D28_27 | 1 | 6 | 123 | 68 | Ascomycota (A) | EF029228.1 | *Hemibeltrania mitrata* | 329 | 43 % | 94 % | HQ445274 |
|  | N3D28_20 | 1 | 7 | 124 | 69 | Mortierellales (Z) | AJ878782.1 | *Mortierella macrocystis* | 691 | 97 % | 89 % | HQ445268 |
|  | N3D28_24 | 1 | 8 | 125 | 60 | Lecanorales (A) | AY756496.1 | *Psilolechia leprosa* | 488 | 88 % | 84 % | HQ445272 |
|  | N3D28_21 | 1 | 9 | 126 | 70 | Botryosphaeriales (A) | EF118042.1 | *Pseudofusicoccum stromaticum* | 347 | 92 % | 78 % | HQ445269 |
|  | N3D28_14 | 1 | 10 | 127 | 48 | Helotiales (A) | AY805585.1 | *Cadophora malorum* | 719 | 90 % | 96 % | HQ445262 |
|  | N3D28_3 | 1 | 11 | 128 | 71 | Dothidiomycetes (A) | DQ529303.1 | *Pseudeurotium bakeri* | 372 | 88 % | 83 % | HQ445253 |
|  | N3D28_15 | 1 | 12 | 129 | 4 | Helotiales (A) | AY347393.1 | *Phialocephala letzii* | 852 | 100 % | 98 % | HQ445263 |
| S1D17 | S1D17_4 | 7 | 1 | 130 | 72 | Agaricales (B) | 4_S1F33_a | *Hebeloma alpinum* | 1189 | 100 % | 99 % | HQ445300 |
|  | S1D17_13 | 1 | 2 | 131 | 72 | Agaricales (B) | UDB002445 | *Hebeloma velutipes* | 1191 | 100 % | 99 % | HQ445306 |
|  | S1D17_20 | 1 | 3 | 132 | 72 | Agaricales (B) | UDB002445 | *Hebeloma velutipes* | 1191 | 100 % | 99 % | HQ445311 |
|  | S1D17_14 | 8 | 4 | 133 | 72 | Agaricales (B) | UDB002445 | *Hebeloma velutipes* | 1215 | 100 % | 99 % | HQ445307 |
|  | S1D17_8 | 1 | 5 | 51 | 22 | Thelephorales (B) | AJ889981.1 | *Tomentella bryophila* | 834 | 100 % | 91 % | HQ445302 |
|  | S1D17_30 | 1 | 6 | 134 | 73 | Thelephorales (B) | AY010275.1 | *Tomentella subclavigera* | 850 | 100 % | 92 % | HQ445320 |
|  | S1D17_7 | 1 | 7 | 135 | 74 | Helotiales (A) | AF486133.1 | *Leptodontidium orchidicola* | 949 | 100 % | 98 % | HQ445301 |
|  | S1D17_18 | 1 | 8 | 136 | 75 | Helotiales (A) | EF093158.1 | *Acephala applanata* | 697 | 100 % | 92 % | HQ445309 |
|  | S1D17_22 | 1 | 9 | 137 | 76 | Russulales (B) | 1_S1F14_a | *Russula delica* | 1223 | 100 % | 100 % | HQ445312 |
|  | S1D17_31 | 1 | 10 | 138 | 77 | Agaricales (B) | AJ494835.1 | *Hypsizygus marmoreus* | 428 | 81 % | 80 % | HQ445321 |
|  | S1D17_32 | 1 | 11 | 114 | 63 | Helotiales (A) | AF169309.1 | *Hymenoscyphus monotropae* | 664 | 100 % | 91 % | HQ445322 |
| S1D23 | S1D23_12 | 2 | 1 | 139 | 78 | Thelephorales (B) | UDB000775 | *Thelephora penicillata* | 904 | 100 % | 94 % | HQ445334 |
|  | S1D23_2 | 2 | 2 | 140 | 78 | Thelephorales (B) | UDB000775 | *Thelephora penicillata* | 920 | 100 % | 94 % | HQ445324 |
|  | S1D23_1 | 6 | 3 | 141 | 78 | Thelephorales (B) | UDB000775 | *Thelephora penicillata* | 928 | 100 % | 95 % | HQ445323 |
|  | S1D23_5 | 2 | 4 | 142 | 79 | Pezizales (A) | EU669387.1 | *Pseudaleuria quinaultiana* | 394 | 68 % | 86 % | HQ445327 |
|  | S1D23_6 | 2 | 5 | 137 | 76 | Russulales (B) | 1_S1F14_a | *Russula delica* | 1218 | 100 % | 99 % | HQ445328 |
|  | S1D23_9 | 2 | 6 | 143 | 39 | Thelephorales (B) | DQ974776.1 | *Tomentella fuscocinerea* | 908 | 100 % | 94 % | HQ445331 |
|  | S1D23_23 | 2 | 7 | 144 | 39 | Thelephorales (B) | DQ974776.1 | *Tomentella fuscocinerea* | 897 | 100 % | 93 % | HQ445343 |
|  | S1D23_13 | 2 | 8 | 145 | 39 | Thelephorales (B) | DQ974776.1 | *Tomentella fuscocinerea* | 901 | 100 % | 94 % | HQ445335 |
|  | S1D23_7 | 1 | 9 | 146 | 80 | Helotiales (A) | DQ320128.1 | *Cadophora finlandia* | 818 | 100 % | 98 % | HQ445329 |
|  | S1D23_27 | 1 | 10 | 147 | 81 | Sebacinales (B) | AJ966754.1 | *Sebacina epigaea* | 756 | 98 % | 92 % | HQ445346 |
|  | S1D23_20 | 1 | 11 | 80 | 43 | Thelephorales (B) | AY010275.1 | *Tomentella subclavigera* | 935 | 100 % | 95 % | HQ445340 |
|  | S1D23_21 | 1 | 12 | 148 | 30 | Thelephorales (B) | DQ974776.1 | *Tomentella fuscocinerea* | 939 | 100 % | 95 % | HQ445341 |
| S2D16 | S2D16_13 | 11 | 1 | 149 | 76 | Russulales (B) | 1_S1F14_a | *Russula delica* | 1215 | 100 % | 99 % | HQ445356 |
|  | S2D16_24 | 2 | 2 | 150 | 82 | Thelephorales (B) | EU819444.1 | *Thelephora terrestris* | 852 | 100 % | 92 % | HQ445361 |
|  | S2D16_21 | 1 | 3 | 151 | 82 | Thelephorales (B) | EU819444.1 | *Thelephora terrestris* | 859 | 100 % | 92 % | HQ445359 |
|  | S2D16_5 | 2 | 4 | 152 | 82 | Thelephorales (B) | EU819444.1 | *Thelephora terrestris* | 848 | 100 % | 92 % | HQ445350 |
|  | S2D16_1 | 7 | 5 | 153 | 82 | Thelephorales (B) | EU819444.1 | *Thelephora terrestris* | 847 | 100 % | 92 % | HQ445347 |
|  | S2D16_10 | 1 | 6 | 154 | 80 | Helotiales (A) | DQ320128.1 | *Cadophora finlandia* | 809 | 100 % | 97 % | HQ445353 |
| S2D33 | S2D33_24 | 1 | 1 | 155 | 83 | Agaricales (B) | DQ384588.1 | *Mycena* cf. *vitilis* | 686 | 96 % | 82 % | HQ445394 |
|  | S2D33_5 | 1 | 2 | 156 | 83 | Agaricales (B) | DQ384588.1 | *Mycena* cf. *vitilis* | 682 | 96 % | 81 % | HQ445375 |
|  | S2D33_15 | 2 | 3 | 157 | 83 | Agaricales (B) | EF530930.1 | *Mycena* cf. *galericulata* | 695 | 96 % | 82 % | HQ445385 |
|  | S2D33_4 | 9 | 4 | 158 | 83 | Agaricales (B) | EF530930.1 | *Mycena* cf. *galericulata* | 695 | 96 % | 82 % | HQ445374 |
|  | S2D33_7 | 1 | 5 | 159 | 84 | Capnodiales (A) | EU019299.1 | *Trimmatostroma betulinum* | 949 | 100 % | 94 % | HQ445377 |
|  | S2D33_21 | 2 | 6 | 160 | 85 | Agaricales (B) | DQ490645.1 | *Mycena amicta* | 334 | 48 % | 89 % | HQ445391 |
|  | S2D33_8 | 2 | 7 | 161 | 4 | Helotiales (A) | FJ031032.1 | *Phialocephala fortinii* | 872 | 100 % | 99 % | HQ445378 |
|  | S2D33_14 | 2 | 8 | 162 | 86 | Thelephorales (B) | UDB000961 | *Tomentella badia* | 1005 | 93 % | 98 % | HQ445384 |
|  | S2D33_2 | 1 | 9 | 163 | 87 | Agaricales (B) | UDB002168 | *Cortinarius anomalus* | 1164 | 100 % | 99 % | HQ445372 |
|  | S2D33_1 | 2 | 10 | 164 | 28 | Helotiales (A) | AF486119.1 | *Phialophora finlandia* | 803 | 100 % | 97 % | HQ445371 |
|  | S2D33_20 | 1 | 11 | 165 | 88 | Helotiales (A) | EU998929.1 | *Articulospora tetracladia* | 159 | 20 % | 98 % | HQ445390 |
| S3D6 | S3D6_14 | 2 | 1 | 166 | 89 | Helotiales (A) | AY348582.1 | *Hymenoscyphus* cf. *fructigenus* | 765 | 99 % | 96 % | HQ445432 |
|  | S3D6_7 | 3 | 2 | 110 | 61 | Agaricales (B) | 17_N1F25_1 | *Cortinarius polaris* | 1201 | 100 % | 100 % | HQ445425 |
|  | S3D6_19 | 1 | 3 | 167 | 8 | Agaricales (B) | AJ889974.1 | *Cortinarius paleaceus* | 861 | 100 % | 97 % | HQ445437 |
|  | S3D6_8 | 1 | 4 | 168 | 4 | Helotiales (A) | AF214579.1 | *Phialocephala fortinii* | 861 | 100 % | 99 % | HQ445426 |
|  | S3D6_1 | 1 | 5 | 169 | 4 | Helotiales (A) | AF214581.1 | *Phialocephala fortinii* | 865 | 100 % | 99 % | HQ445419 |
|  | S3D6_6 | 2 | 6 | 170 | 28 | Helotiales (A) | AF486119.1 | *Phialophora finlandia* | 803 | 100 % | 97 % | HQ445424 |
|  | S3D6_13 | 1 | 7 | 116 | 28 | Helotiales (A) | AF486119.1 | *Phialophora finlandia* | 814 | 100 % | 98 % | HQ445431 |
|  | S3D6_22 | 4 | 8 | 41 | 6 | Dothideomycetes (A) | AY940649.1 | *Cenococcum geophilum* | 818 | 100 % | 100 % | HQ445440 |
|  | S3D6_5 | 1 | 9 | 171 | 90 | Dothideomycetes (A) | AY916479.1 | *Helicosporium guianense* | 304 | 43 % | 94 % | HQ445423 |
|  | S3D6_16 | 1 | 10 | 172 | 91 | Teloschistales (A) | AY081157.1 | *Xanthomendoza montana* | 304 | 52 % | 88 % | HQ445434 |
|  | S3D6_12 | 1 | 11 | 173 | 92 | Helotiales (A) | EF413597.1 | *Cryptosporiopsis radicicola* | 854 | 100 % | 99 % | HQ445430 |
|  | S3D6_4 | 1 | 12 | 174 | 93 | Mortierellales (Z) | AJ878782.1 | *Mortierella macrocystis* | 205 | 21 % | 88 % | HQ445422 |
|  | S3D6_11 | 1 | 13 | 175 | 94 | Helotiales (A) | EU272533.1 | *Leptodontidium orchidicola* | 854 | 100 % | 94 % | HQ445429 |
|  | S3D6_23 | 1 | 14 | 176 | 95 | Helotiales (A) | DQ431180.1 | *Hymenoscyphus epiphyllus* | 787 | 100 % | 97 % | HQ445441 |
|  | S3D6_15 | 1 | 15 | 177 | 80 | Helotiales (A) | DQ320128.1 | *Cadophora finlandia* | 800 | 100 % | 97 % | HQ445433 |
|  | S3D6_18 | 1 | 16 | 178 | 96 | Acarosporales (A) | DQ374141.1 | *Acarospora smaragdula* | 354 | 76 % | 81 % | HQ445436 |
|  | S3D6_20 | 1 | 17 | 179 | 97 | Leotiomycetes (A) | EU678379.1 | *Leohumicola verrucosa* | 336 | 57 % | 88 % | HQ445438 |
| S3D35 | S3D35_6 | 5 | 1 | 180 | 4 | Helotiales (A) | EF093162.1 | *Phialocephala fortinii* | 850 | 100 % | 98 % | HQ445398 |
|  | S3D35_23 | 2 | 2 | 181 | 76 | Russulales (B) | 1_S1F14_a | *Russula delica* | 1191 | 100 % | 99 % | HQ445409 |
|  | S3D35_31 | 2 | 3 | 182 | 76 | Russulales (B) | 1_S1F14_a | *Russula delica* | 1202 | 100 % | 99 % | HQ445415 |
|  | S3D35_15 | 1 | 4 | 183 | 76 | Russulales (B) | 1_S1F14_a | *Russula delica* | 1191 | 100 % | 99 % | HQ445403 |
|  | S3D35_24 | 1 | 5 | 184 | 76 | Russulales (B) | 1_S1F14_a | *Russula delica* | 1183 | 100 % | 99 % | HQ445410 |
|  | S3D35_5 | 1 | 6 | 185 | 76 | Russulales (B) | 1_S1F14_a | *Russula delica* | 1191 | 100 % | 99 % | HQ445397 |
|  | S3D35_25 | 4 | 7 | 186 | 76 | Russulales (B) | 1_S1F14_a | *Russula delica* | 1215 | 100 % | 99 % | HQ445411 |
|  | S3D35_29 | 6 | 8 | 137 | 76 | Russulales (B) | 1_S1F14_a | *Russula delica* | 1223 | 100 % | 99 % | HQ445414 |
|  | S3D35_22 | 1 | 9 | 187 | 80 | Helotiales (A) | DQ320128.1 | *Cadophora finlandia* | 800 | 100 % | 97 % | HQ445408 |
|  | S3D35_28 | 1 | 10 | 188 | 67 | Teloschistales (A) | AY233218.1 | *Caloplaca thallincola* | 306 | 48 % | 91 % | HQ445413 |
| T1D4 | T1D4_2_2 | 2 | 1 | 189 | 98 | Saccharomycetales (A) | DQ911450.1 | *Candida novakii* | 206 | 43 % | 86 % | HQ445475 |
|  | T1D4_2_4 | 3 | 2 | 190 | 99 | Chaetothyriales (A) | AY213652.1 | *Exophiala salmonis* | 877 | 100 % | 95 % | HQ445477 |
|  | T1D4_2_1 | 2 | 3 | 191 | 100 | Sebacinales (B) | AJ966754.1 | *Sebacina epigaea* | 749 | 98 % | 92 % | HQ445474 |
|  | T1D4_2_6 | 1 | 4 | 192 | 100 | Sebacinales (B) | AJ966754.1 | *Sebacina epigaea* | 731 | 98 % | 91 % | HQ445479 |
|  | T1D4_2_18 | 2 | 5 | 193 | 101 | Sebacinales (B) | AJ966754.1 | *Sebacina epigaea* | 764 | 98 % | 93 % | HQ445489 |
|  | T1D4_2_10 | 1 | 6 | 194 | 102 | Thelephorales (B) | DQ068972.1 | *Tomentella lilacinogrisea* | 841 | 100 % | 91 % | HQ445483 |
|  | T1D4_4 | 1 | 7 | 195 | 102 | Thelephorales (B) | DQ068972.1 | *Tomentella lilacinogrisea* | 850 | 100 % | 92 % | HQ445470 |
|  | T1D4_2_14 | 1 | 8 | 196 | 103 | Thelephorales (B) | DQ974776.1 | *Tomentella fuscocinerea* | 856 | 100 % | 92 % | HQ445485 |
|  | T1D4_2_17 | 1 | 9 | 197 | 104 | Thelephorales (B) | DQ974771.1 | *Thelephora anthocephala* | 893 | 100 % | 93 % | HQ445488 |
|  | T1D4_5 | 1 | 10 | 198 | 105 | Helotiales (A) | DQ404350.1 | *Cadophora malorum* | 646 | 91 % | 93 % | HQ445471 |
|  | T1D4_2_19 | 1 | 11 | 199 | 67 | Teloschistales (A) | EU639619.1 | *Caloplaca xantholyta* | 304 | 39 % | 97 % | HQ445490 |
|  | T1D4_2_7 | 1 | 12 | 200 | 106 | Agaricales (B) | DQ490623.1 | *Hemimycena gracilis* | 547 | 92 % | 82 % | HQ445480 |
|  | T1D4_8 | 1 | 13 | 117 | 27 | Teloschistales (A) | EU639615.1 | *Teloschistes lacunosus* | 313 | 45 % | 95 % | HQ445473 |
|  | T1D4_2 | 1 | 14 | 201 | 107 | Thelephorales (B) | AJ889981.1 | *Tomentella bryophila* | 892 | 100 % | 93 % | HQ445468 |
|  | T1D4_3 | 1 | 15 | 202 | 108 | Agaricales (B) | 32_N3F21 | *Mycena* cf. *galopus* | 1104 | 100 % | 98 % | HQ445469 |
|  | T1D4_7 | 1 | 16 | 203 | 109 | Teloschistales (A) | EF643516.1 | *Caloplaca ammiospila* | 919 | 98 % | 99 % | HQ445472 |
|  | T1D4_2_3 | 1 | 17 | 204 | 110 | Helotiales (A) | DQ202516.1 | *Varicosporium delicatum* | 672 | 63 % | 94 % | HQ445476 |
|  | T1D4_2_15 | 1 | 18 | 205 | 57 | Leotiomycetes (A) | AY706325.1 | *Leohumicola verrucosa* | 733 | 100 % | 94 % | HQ445486 |
|  | T1D4_2_16 | 1 | 19 | 206 | 111 | Helotiales (A) | DQ068996.1 | *Tetracladium maxilliforme* | 854 | 100 % | 100 % | HQ445487 |
| T1D26 | T1D26_16 | 1 | 1 | 207 | 83 | Agaricales (B) | EU846300.1 | *Mycena hudsoniana* | 779 | 78 % | 92 % | HQ445457 |
|  | T1D26_13 | 1 | 2 | 208 | 83 | Agaricales (B) | EF530930.1 | *Mycena* cf. *galericulata* | 700 | 96 % | 82 % | HQ445454 |
|  | T1D26_9 | 1 | 3 | 209 | 83 | Agaricales (B) | EU846300.1 | *Mycena hudsoniana* | 765 | 78 % | 91 % | HQ445462 |
|  | T1D26_25 | 1 | 4 | 210 | 83 | Agaricales (B) | EU846300.1 | *Mycena hudsoniana* | 770 | 78 % | 92 % | HQ445466 |
|  | T1D26_20 | 1 | 5 | 211 | 83 | Agaricales (B) | EF530930.1 | *Mycena* cf. *galericulata* | 691 | 96 % | 82 % | HQ445464 |
|  | T1D26_18 | 7 | 6 | 212 | 83 | Agaricales (B) | EF530930.1 | *Mycena* cf. *galericulata* | 706 | 96 % | 82 % | HQ445463 |
|  | T1D26_8 | 2 | 7 | 213 | 6 | Dothideomycetes (A) | EU427331.1 | *Cenococcum geophilum* | 801 | 100 % | 99 % | HQ445450 |
|  | T1D26_14 | 1 | 8 | 214 | 4 | Helotiales (A) | FJ031032.1 | *Phialocephala fortinii* | 872 | 100 % | 99 % | HQ445455 |
|  | T1D26_17 | 1 | 9 | 215 | 4 | Helotiales (A) | FJ031032.1 | *Phialocephala fortinii* | 868 | 100 % | 99 % | HQ445458 |
|  | T1D26_5 | 2 | 10 | 216 | 112 | Thelephorales (B) | UDB000952 | *Tomentella badia* | 1076 | 92 % | 99 % | HQ445447 |
|  | T1D26_2 | 1 | 11 | 217 | 113 | Helotiales (A) | AY789318.1 | *Geoglossum glabrum* | 560 | 93 % | 87 % | HQ445444 |
|  | T1D26_15 | 1 | 12 | 218 | 114 | Pleosporales (A) | DQ018090.1 | *Dictyosporium heptasporum* | 466 | 87 % | 84 % | HQ445456 |
|  | T1D26_3 | 1 | 13 | 219 | 115 | Agaricales (B) | AM882714.1 | *Inocybe egenula* | 881 | 98 % | 95 % | HQ445445 |
|  | T1D26_7 | 1 | 14 | 220 | 116 | Helotiales (A) | EF029215.1 | *Spirosphaera beverwijkiana* | 663 | 84 % | 93 % | HQ445449 |
|  | T1D26_10 | 1 | 15 | 221 | 117 | Sebacinales (B) | DQ520096.1 | *Serendipita vermifera* | 508 | 100 % | 80 % | HQ445451 |
|  | T1D26_11 | 1 | 16 | 222 | 118 | Agaricales (B) | AY312986.1 | *Hebeloma saliciphilum* | 1090 | 100 % | 99 % | HQ445452 |
| T2D2 | T2D2_6 | 5 | 1 | 223 | 119 | Russulales (B) | UDB000312 | *Lactarius aurantiacus* | 1172 | 100 % | 98 % | HQ445520 |
|  | T2D2_25 | 5 | 2 | 224 | 120 | Agaricales (B) | AM882711.1 | *Inocybe subexilis* | 747 | 94 % | 94 % | HQ445536 |
|  | T2D2_1 | 2 | 3 | 225 | 121 | Thelephorales (B) | AY010277.1 | *Tomentella stuposa* | 821 | 100 % | 91 % | HQ445515 |
|  | T2D2_27 | 1 | 4 | 226 | 122 | Thelephorales (B) | EF644115.1 | *Tomentella atramentaria* | 1011 | 96 % | 99 % | HQ445538 |
|  | T2D2_19 | 1 | 5 | 227 | 123 | Thelephorales (B) | UDB000961 | *Tomentella badia* | 922 | 93 % | 96 % | HQ445530 |
|  | T2D2_3 | 2 | 6 | 228 | 124 | Russulales (B) | UDB001641 | *Russula versicolor* | 1201 | 100 % | 99 % | HQ445517 |
|  | T2D2_26 | 1 | 7 | 229 | 6 | Dothideomycetes (A) | DQ068980.1 | *Cenococcum geophilum* | 810 | 100 % | 99 % | HQ445537 |
|  | T2D2_9 | 1 | 8 | 230 | 6 | Dothideomycetes (A) | AF495462.1 | *Cenococcum geophilum* | 796 | 100 % | 99 % | HQ445523 |
|  | T2D2_4 | 3 | 9 | 231 | 125 | Helotiales (A) | EF029215.1 | *Spirosphaera beverwijkiana* | 695 | 89 % | 94 % | HQ445518 |
|  | T2D2_10 | 1 | 10 | 232 | 126 | Dothideomycetes (A) | EU030274.1 | *Seifertia azaleae* | 700 | 98 % | 95 % | HQ445524 |
|  | T2D2_11 | 1 | 11 | 233 | 127 | Sebacinales (B) | DQ520096.1 | *Serendipita vermifera* | 506 | 100 % | 80 % | HQ445525 |
|  | T2D2_22 | 1 | 12 | 234 | 128 | Pezizales (A) | AJ969622.2 | *Genea hispidula* | 306 | 35 % | 89 % | HQ445533 |
| T2D15 | T2D15_2 | 1 | 1 | 235 | 83 | Agaricales (B) | EF530930.1 | *Mycena* cf. *galericulata* | 702 | 95 % | 82 % | HQ445492 |
|  | T2D15_22 | 1 | 2 | 236 | 83 | Agaricales (B) | EF530930.1 | *Mycena* cf. *galericulata* | 693 | 95 % | 82 % | HQ445511 |
|  | T2D15_25 | 2 | 3 | 237 | 119 | Russulales (B) | UDB000312 | *Lactarius aurantiacus* | 1144 | 100 % | 97 % | HQ445513 |
|  | T2D15_6 | 1 | 4 | 238 | 119 | Russulales (B) | UDB000312 | *Lactarius aurantiacus* | 1134 | 100 % | 97 % | HQ445496 |
|  | T2D15_21 | 7 | 5 | 239 | 119 | Russulales (B) | UDB000312 | *Lactarius aurantiacus* | 1164 | 100 % | 97 % | HQ445510 |
|  | T2D15_16 | 2 | 6 | 240 | 4 | Helotiales (A) | FJ031032.1 | *Phialocephala fortinii* | 868 | 100 % | 99 % | HQ445505 |
|  | T2D15_3 | 1 | 7 | 241 | 4 | Helotiales (A) | FJ031032.1 | *Phialocephala fortinii* | 859 | 100 % | 99 % | HQ445493 |
|  | T2D15_8 | 2 | 8 | 161 | 4 | Helotiales (A) | FJ031032.1 | *Phialocephala fortinii* | 872 | 100 % | 99 % | HQ445498 |
|  | T2D15_1 | 1 | 9 | 242 | 28 | Helotiales (A) | AF486119.1 | *Phialophora finlandia* | 816 | 99 % | 98 % | HQ445491 |
|  | T2D15_10 | 1 | 10 | 243 | 28 | Helotiales (A) | AF486119.1 | *Phialophora finlandia* | 823 | 100 % | 98 % | HQ445500 |
|  | T2D15_12 | 1 | 11 | 244 | 129 | Helotiales (A) | AY781230.1 | *Leptodontidium elatius* | 673 | 98 % | 91 % | HQ445501 |
|  | T2D15_13 | 1 | 12 | 245 | 130 | Eurotiales (A) | AF033488.1 | *Penicillium soppii* | 906 | 100 % | 100 % | HQ445502 |
|  | T2D15_5 | 1 | 13 | 246 | 131 | Sebacinales (B) | AF490393.1 | *Sebacina* aff. *epigaea* | 616 | 100 % | 85 % | HQ445495 |
|  | T2D15_14 | 1 | 14 | 247 | 6 | Dothideomycetes (A) | AF495462.1 | *Cenococcum geophilum* | 800 | 100 % | 99 % | HQ445503 |
|  | T2D15_18 | 1 | 15 | 248 | 132 | Dothideomycetes (A) | AY916453.1 | *Helicoma muelleri* | 315 | 48 % | 93 % | HQ445507 |
| T3D23 | T3D23_12 | 8 | 1 | 249 | 83 | Agaricales (B) | DQ384588.1 | *Mycena* cf. *vitilis* | 708 | 95 % | 82 % | HQ445572 |
|  | T3D23_5 | 2 | 2 | 250 | 80 | Helotiales (A) | DQ320128.1 | *Cadophora finlandia* | 798 | 100 % | 97 % | HQ445567 |
|  | T3D23_9 | 2 | 3 | 251 | 28 | Helotiales (A) | AF486119.1 | *Phialophora finlandia* | 830 | 100 % | 98 % | HQ445569 |
|  | T3D23_3 | 5 | 4 | 252 | 80 | Helotiales (A) | DQ320128.1 | *Cadophora finlandia* | 810 | 100 % | 97 % | HQ445565 |
|  | T3D23_28 | 1 | 5 | 253 | 80 | Helotiales (A) | DQ320128.1 | *Cadophora finlandia* | 794 | 100 % | 97 % | HQ445585 |
|  | T3D23_24 | 1 | 6 | 254 | 133 | Diversisporales (G) | AF133776.1 | *Acaulospora colossica spore* | 140 | 28 % | 83 % | HQ445581 |
|  | T3D23_27 | 1 | 7 | 255 | 134 | Helotiales (A) | DQ320128.1 | *Cadophora finlandia* | 722 | 100 % | 94 % | HQ445584 |
|  | T3D23_2 | 1 | 8 | 256 | 132 | Dothideomycetes (A) | AY916453.1 | *Helicoma muelleri* | 309 | 45 % | 94 % | HQ445564 |
|  | T3D23_14 | 1 | 9 | 257 | 135 | Helotiales (A) | EF029228.1 | *Hemibeltrania mitrata* | 410 | 63 % | 89 % | HQ445574 |
|  | T3D23_10 | 1 | 10 | 258 | 136 | Agaricales (B) | EU118617.1 | *Clavulinopsis helvola* | 399 | 95 % | 78 % | HQ445570 |
|  | T3D23_11 | 1 | 11 | 259 | 137 | Sebacinales (B) | DQ520096.1 | *Serendipita vermifera* | 499 | 100 % | 80 % | HQ445571 |
| T3D16 | T3D16_9 | 1 | 1 | 260 | 138 | Cantharellales (B) | AY082606.1 | *Craterellus lutescens* | 1225 | 100 % | 99 % | HQ445542 |
|  | T3D16_2_7 | 1 | 2 | 261 | 138 | Cantharellales (B) | AY082606.1 | *Craterellus lutescens* | 1232 | 100 % | 99 % | HQ445558 |
|  | T3D16_1 | 2 | 3 | 262 | 138 | Cantharellales (B) | AY082606.1 | *Craterellus lutescens* | 1238 | 100 % | 99 % | HQ445539 |
|  | T3D16_2_11 | 1 | 4 | 263 | 138 | Cantharellales (B) | AY082606.1 | *Craterellus lutescens* | 1218 | 100 % | 99 % | HQ445562 |
|  | T3D16_18 | 1 | 5 | 264 | 138 | Cantharellales (B) | AY082606.1 | *Craterellus lutescens* | 1223 | 100 % | 99 % | HQ445545 |
|  | T3D16_2_1 | 1 | 6 | 265 | 138 | Cantharellales (B) | AY082606.1 | *Craterellus lutescens* | 1229 | 100 % | 99 % | HQ445553 |
|  | T3D16_30 | 3 | 7 | 266 | 138 | Cantharellales (B) | AY082606.1 | *Craterellus lutescens* | 1236 | 100 % | 99 % | HQ445552 |
|  | T3D16_27 | 3 | 8 | 267 | 138 | Cantharellales (B) | AY082606.1 | *Craterellus lutescens* | 1234 | 100 % | 99 % | HQ445550 |
|  | T3D16_2_2 | 8 | 9 | 268 | 138 | Cantharellales (B) | AY082606.1 | *Craterellus lutescens* | 1243 | 100 % | 100 % | HQ445554 |
|  | T3D16_2_4 | 2 | 10 | 161 | 4 | Helotiales (A) | EF446148.1 | *Phialocephala fortinii* | 874 | 100 % | 100 % | HQ445556 |
|  | T3D16_5 | 1 | 11 | 249 | 83 | Agaricales (B) | DQ384588.1 | *Mycena* cf. *vitilis* | 708 | 95 % | 82 % | HQ445540 |
