# Supplementary material for: High diversity of root associated fungi in both alpine and arctic Dryas octopetala
Source: BMC Plant Biol. 2010 Nov 11;10:244. doi: 10.1186/1471-2229-10-244 (PMC3095326; doi:10.1186/1471-2229-10-244)
Supplement: Additional file 3 — List of all detected OTUs. [file 1471-2229-10-244-S3.DOC]

Additional file 3 - List of all detected OTUs

| **OTU#^1^** | **OTU-name** | **Sub-localities^2^** | **#3** | **SS^4^** |
| --- | --- | --- | --- | --- |
| 4 | *Phialocephala fortinii* | (F1,F3),(N3),(S2,S3),(T1,T2,T3) | 34 | 100% |
| 6 | *Cenococcum geophilum* | (F1,F2,F3),(N2),(S3),(T1,T2) | 34 | 100% |
| 28 | *Cadophora finlandia*1 | (F3),(N1,N3),(S2,S3),(T2,T3) | 13 | 98% |
| 8 | *Cortinarius* aff. *inconspicuus/diasemospermus* | (F1,F2,F3),(N1,N2),(S3) | 20 | 99% |
| 22 | *Tomentella* sp.1 | (F2,F3),(N1),(S1) | 8 | 92% |
| 67 | *Caloplaca* sp. | (N3),(S3),(T1) | 4 | 91% |
| 80 | *Cadophora finlandia* 2 | (S1,S2,S3),(T3) | 12 | 98% |
| 33 | Inocybaceae 1 | (F3),(N2) | 12 | 82% |
| 39 | *Tomentella* sp.2 | (N1,N3),(S1) | 9 | 94% |
| 43 | *Tomentella* sp.3 | (N1),(S1) | 13 | 95% |
| 61 | *Cortinarius* aff. *polaris* | (N3),(S3) | 13 | 100% |
| 63 | *Hymenoscyphus* sp.1 | (N3),(S1) | 3 | 91% |
| 83 | Mycenaceae 1 | (S2),(T1,T2,T3) | 36 | 82% |
| 2 | *Hebeloma* aff. *alpinum* | (F1),(N1,N2) | 11 | 99% |
| 57 | *Leohumicola* sp.1 | (N2),(T1) | 2 | 94% |
| 30 | *Tomentella* sp.7 | (F3),(S1) | 3 | 95% |
| 47 | *Cortinarius* aff. *tenebricus* | (N1,N3) | 11 | 100% |
| 76 | *Russula delica* | (S1,S2,S3) | 31 | 100% |
| 48 | *Cadophora* sp.1 | (N1,N2,N3) | 5 | 96% |
| 40 | *Tomentella* sp.4 | (N1,N2) | 4 | 93% |
| 72 | *Hebeloma* aff. *velutipes* | (S1) | 17 | 99% |
| 15 | *Sebacina incrustans* | (F2) | 2 | 100% |
| 25 | *Cortinarius decipiens* | (F2) | 5 | 99% |
| 138 | *Craterellus lutescens* | (T3) | 20 | 100% |
| 27 | *Teloschistes sp.* | (F2),(N3),(T1) | 3 | 95% |
| 17 | *Inocybe nitidiuscula* | (F2) | 9 | 99% |
| 23 | *Russula* sp. | (F2) | 10 | 90% |
| 78 | *Thelephora* sp.1 | (S1) | 10 | 94% |
| 82 | *Thelephora* sp.2 | (S2) | 12 | 92% |
| 119 | *Lactarius luculentus/aurantiacus* | (T2) | 15 | 98% |
| 42 | Cantharellales 1 | (N1) | 5 | 79% |
| 60 | Lecanorales | (N3) | 3 | 84% |
| 50 | *Thelephora* sp.3 | (N2) | 3 | 94% |
| 102 | *Tomentella* sp.5 | (T1) | 2 | 92% |
| 21 | *Cordyceps sinensis*/*Coniochaeta ligniaria* | (F2),(N3) | 2 | 91% |
| 37 | *Hebeloma* sp. | (N1) | 3 | 97% |
| 52 | *Tomentella* sp.6 | (N2) | 3 | 94% |
| 100 | *Sebacina* sp.1 | (T1) | 3 | 92% |
| 18 | Inocybaceae sp.2 | (F2) | 4 | 86% |
| 132 | *Helicoma* sp. | (T2),(T3) | 2 | 93% |
| 7 | *Lunulospora curvula* | (F1) | 2 | 97% |
| 16 | *Clavulina* sp.1 | (F2) | 2 | 93% |
| 1 | Cantharellales 2 | (F1) | 1 | 84% |
| 3 | *Phialocephala helvetica* | (F1) | 1 | 97% |
| 5 | *Trichophaea* sp. | (F1) | 1 | 95% |
| 9 | *Inocybe* sp.3 | (F1) | 1 | 95% |
| 10 | *Tomentella* sp.8 | (F1) | 1 | 95% |
| 11 | *Sistotrema* sp. | (F1) | 1 | 96% |
| 12 | *Lachnum pygmaeum* | (F1) | 1 | 98% |
| 13 | *Sebacina* sp.2 | (F1) | 1 | 90% |
| 14 | *Leohumicola minima*1 | (F1) | 1 | 90% |
| 19 | *Tomentella* sp.9 | (F2) | 1 | 93% |
| 20 | *Gyoerffyella*sp. | (F2) | 1 | 94% |
| 24 | *Tomentella* sp.10 | (F2) | 1 | 94% |
| 26 | *Leohumicola minima* sp.2 | (F2) | 1 | 97% |
| 29 | Botryosphaeriaceae 1 | (F3) | 1 | 85% |
| 31 | *Tomentella* sp.11 | (F3) | 1 | 93% |
| 32 | Pseudeurotiaceae 1 | (F3) | 1 | 81% |
| 34 | *Tremellodendron* sp. | (F3) | 1 | 93% |
| 36 | *Sebacina* sp.3 | (N1) | 1 | 95% |
| 38 | *Fusidium* sp. (incerta sedis) | (N1) | 1 | 86% |
| 41 | *Inocybe* cf. *flocculosa* | (N1) | 1 | 98% |
| 44 | *Tomentella* sp.12 | (N1) | 1 | 95% |
| 45 | *Tomentella* sp.13 | (N1) | 1 | 94% |
| 46 | *Tomentella* sp.14 | (N1) | 1 | 92% |
| 49 | Plectosphaerellaceae | (N1) | 1 | 81% |
| 51 | *Inocybe* sp.4 | (N2) | 1 | 95% |
| 53 | *Leohumicola* sp.2 | (N2) | 1 | 90% |
| 54 | Cantharellales 3 | (N2) | 1 | 82% |
| 55 | *Thelephora* sp.4 | (N2) | 1 | 94% |
| 56 | *Inocybe fastigiata* | (N2) | 1 | 100% |
| 58 | *Varicosporium* sp.1 | (N2) | 1 | 94% |
| 59 | *Sarcinomyces* sp. | (N3) | 1 | 92% |
| 62 | *Sebacina* sp.4 | (N3) | 1 | 93% |
| 64 | *Hymenoscyphus* cf. *fructigenus* | (N3) | 1 | 98% |
| 65 | Halosphaeriaceae | (N3) | 1 | 83% |
| 66 | *Cortinarius rubricosus* | (N3) | 1 | 97% |
| 68 | *Hemibeltrania* sp.1 | (N3) | 1 | 94% |
| 69 | Mortierellaceae 1 | (N3) | 1 | 89% |
| 70 | Botryosphaeriaceae 2 | (N3) | 1 | 78% |
| 71 | Pseudeurotiaceae 2 | (N3) | 1 | 83% |
| 73 | *Tomentella* sp.15 | (S1) | 1 | 92% |
| 74 | *Leptodontidium orchidicola* | (S1) | 1 | 98% |
| 75 | *Acephala* sp. | (S1) | 1 | 92% |
| 77 | Lyophyllaceae | (S1) | 1 | 80% |
| 79 | Pyronemataceae | (S1) | 1 | 86% |
| 81 | *Sebacina* sp.5 | (S1) | 1 | 92% |
| 84 | *Trimmatostroma* sp. | (S2) | 1 | 94% |
| 85 | Mycenaceae 2 | (S2) | 1 | 89% |
| 86 | *Tomentella* sp.16 | (S2) | 1 | 98% |
| 87 | *Cortinarius anomalus* | (S2) | 1 | 99% |
| 88 | *Articulospora* sp. | (S2) | 1 | 98% |
| 89 | *Hymenoscyphus* sp.2 | (S3) | 1 | 96% |
| 90 | *Helicosporium* sp. | (S3) | 1 | 94% |
| 91 | Teloschistaceae | (S3) | 1 | 88% |
| 92 | *Cryptosporiopsis radicicola* | (S3) | 1 | 99% |
| 93 | Mortierellaceae 2 | (S3) | 1 | 88% |
| 94 | *Leptodontidium* sp.1 | (S3) | 1 | 94% |
| 95 | *Hymenoscyphus epiphyllus* | (S3) | 1 | 97% |
| 96 | Acarosporaceae | (S3) | 1 | 81% |
| 97 | *Leohumicola* sp.3 (incerta sedis) | (S3) | 1 | 88% |
| 98 | Saccharomycetales | (T1) | 1 | 86% |
| 99 | *Exophiala* sp. | (T1) | 1 | 95% |
| 101 | *Sebacina* sp.6 | (T1) | 1 | 93% |
| 103 | *Tomentella* sp.17 | (T1) | 1 | 92% |
| 104 | *Thelephora* sp.5 | (T1) | 1 | 93% |
| 105 | *Cadophora* sp.2 | (T1) | 1 | 93% |
| 106 | Mycenaceae 3 | (T1) | 1 | 82% |
| 107 | *Tomentella* sp.18 | (T1) | 1 | 93% |
| 108 | *Mycena* cfr. *galopus* | (T1) | 1 | 98% |
| 109 | *Caloplaca ammiospila* | (T1) | 1 | 99% |
| 110 | *Varicosporium* sp.2 | (T1) | 1 | 94% |
| 111 | *Tetracladium maxilliforme* | (T1) | 1 | 100% |
| 112 | *Tomentella* sp.19 | (T1) | 1 | 92% |
| 113 | Geoglossaceae sp. | (T1) | 1 | 87% |
| 114 | Pleosporales sp. | (T1) | 1 | 84% |
| 115 | *Inocybe* sp.5 | (T1) | 1 | 95% |
| 116 | *Spirosphaera* sp.1 | (T1) | 1 | 93% |
| 117 | Auriculariales 1 | (T1) | 1 | 80% |
| 118 | *Hebeloma saliciphilum* | (T1) | 1 | 99% |
| 120 | *Inocybe* sp.6 | (T2) | 1 | 94% |
| 121 | *Tomentella* sp.20 | (T2) | 1 | 91% |
| 122 | *Tomentella* sp.21 | (T2) | 1 | 99% |
| 123 | *Tomentella* sp.22 | (T2) | 1 | 93% |
| 124 | *Russula versicolor* | (T2) | 1 | 99% |
| 125 | *Spirosphaera* sp.2 | (T2) | 1 | 94% |
| 126 | *Seifertia* sp. | (T2) | 1 | 95% |
| 127 | Auriculariales 2 | (T2) | 1 | 80% |
| 128 | Pyronemataceae | (T2) | 1 | 89% |
| 129 | *Leptodontidium* sp.2 | (T2) | 1 | 91% |
| 130 | *Penicillium soppii* | (T2) | 1 | 100% |
| 131 | Sebacinaceae | (T2) | 1 | 85% |
| 133 | Acaulosporaceae | (T3) | 1 | 83% |
| 134 | *Cadophora* sp.3 | (T3) | 1 | 94% |
| 135 | *Hemibeltrania* sp.2 (incerta sedis) | (T3) | 1 | 89% |
| 136 | Clavariaceae | (T3) | 1 | 78% |
| 137 | Auriculariales 3 | (T3) | 1 | 80% |

^1^OTU number. ^2^Main localities; Finse (F), Longyearbyen (S), Ny-Ålesund (N), and Tromsø (T). Sub-localities indicated by numbers 1-3. ^3^The number of clones (C) observed for the respective OTU.  ^4^Sequence similarity (SS) to best Blast match (GenBank/UNITE or own reference sequence).
