# Supplementary material for: High diversity of root associated fungi in both alpine and arctic Dryas octopetala
Source: BMC Plant Biol. 2010 Nov 11;10:244. doi: 10.1186/1471-2229-10-244 (PMC3095326; doi:10.1186/1471-2229-10-244)

**Additional file 4 - phylogenetic trees**

Most parsimonious trees (MPTs) of environmental sequences with affinity to reference sequences of (a) Sebacinaceae, (b) *Hebeloma*, (c) *Cadophora*, (d) *Tomentella*, (e) *Leohumicola*, (f) *Hymenoscyphus*, (g) *Cenococcum geophilum*, (h) *Phialocephala*, (i) *Thelephora*, (j) *Russula*, and (k) *Cortinarius*. The trees includes the obtained environmental sequences (the localities indicated in different colours), accessions from GenBank/UNITE with highest sequence similarity to the environmental sequences, and reference sequences from basidiocarps generated in this study (indicated by a symbol of a fruit body). The grey boxes encompass environmental sequences that belong to the same OTU (singletons OTUs are indicated by small grey boxes). Branches that collapsed in strict consensus trees are indicated by arrows. Number of MPTs and tree lenghts are given for each of the analyses. Jackknife support values are given below branches. Asterisks (*) behind environmental sequences from different root systems indicate 100% similarity.


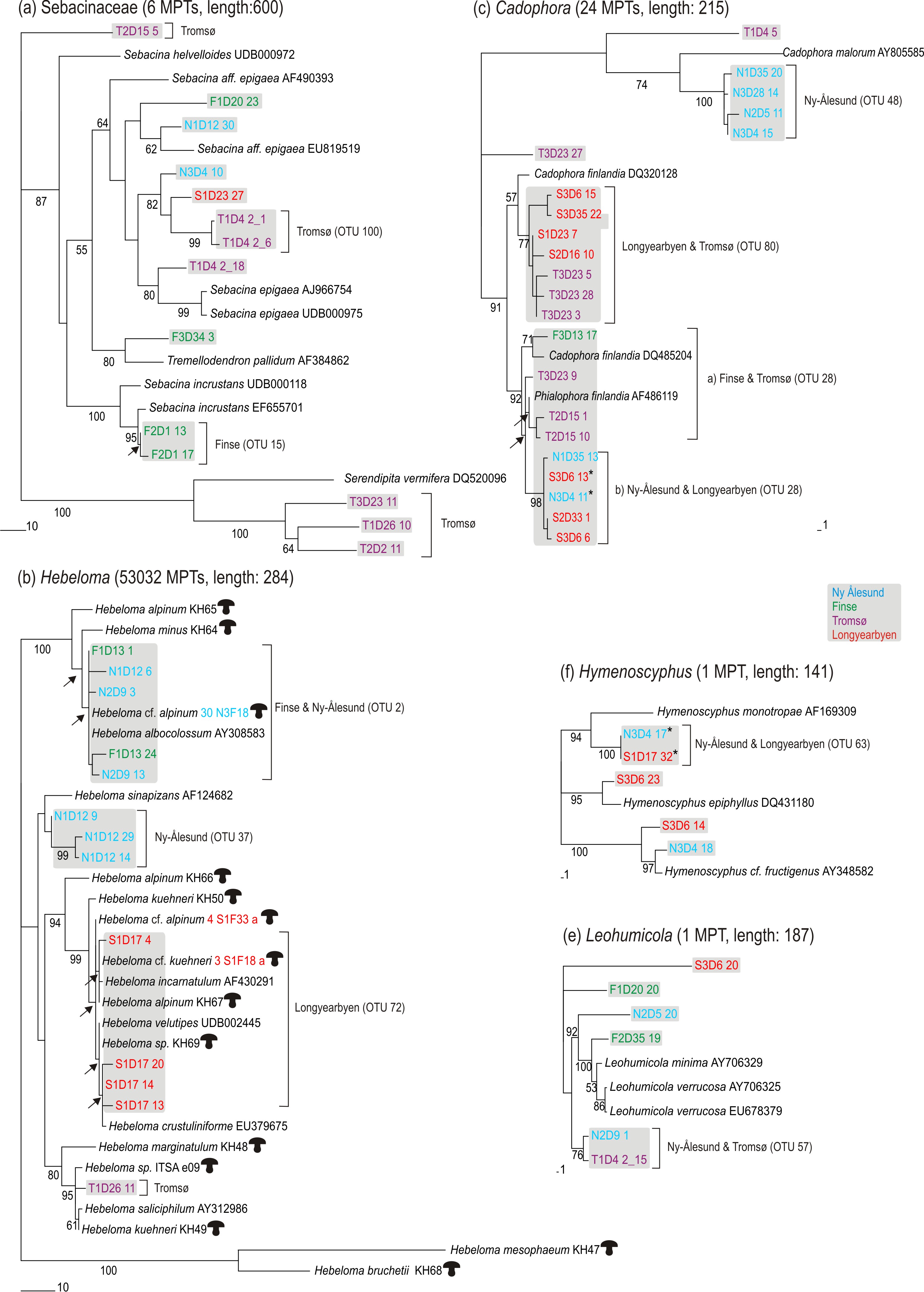

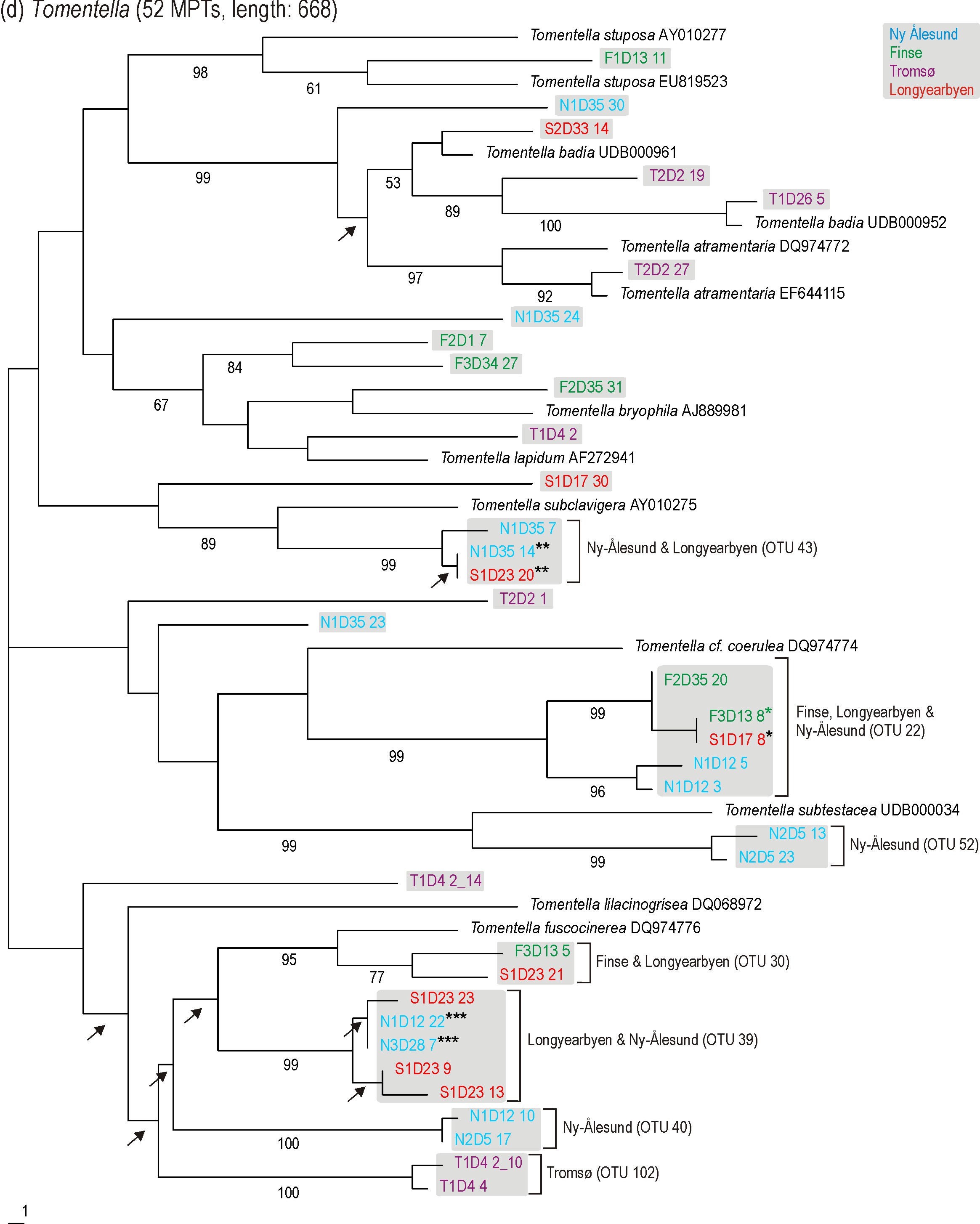

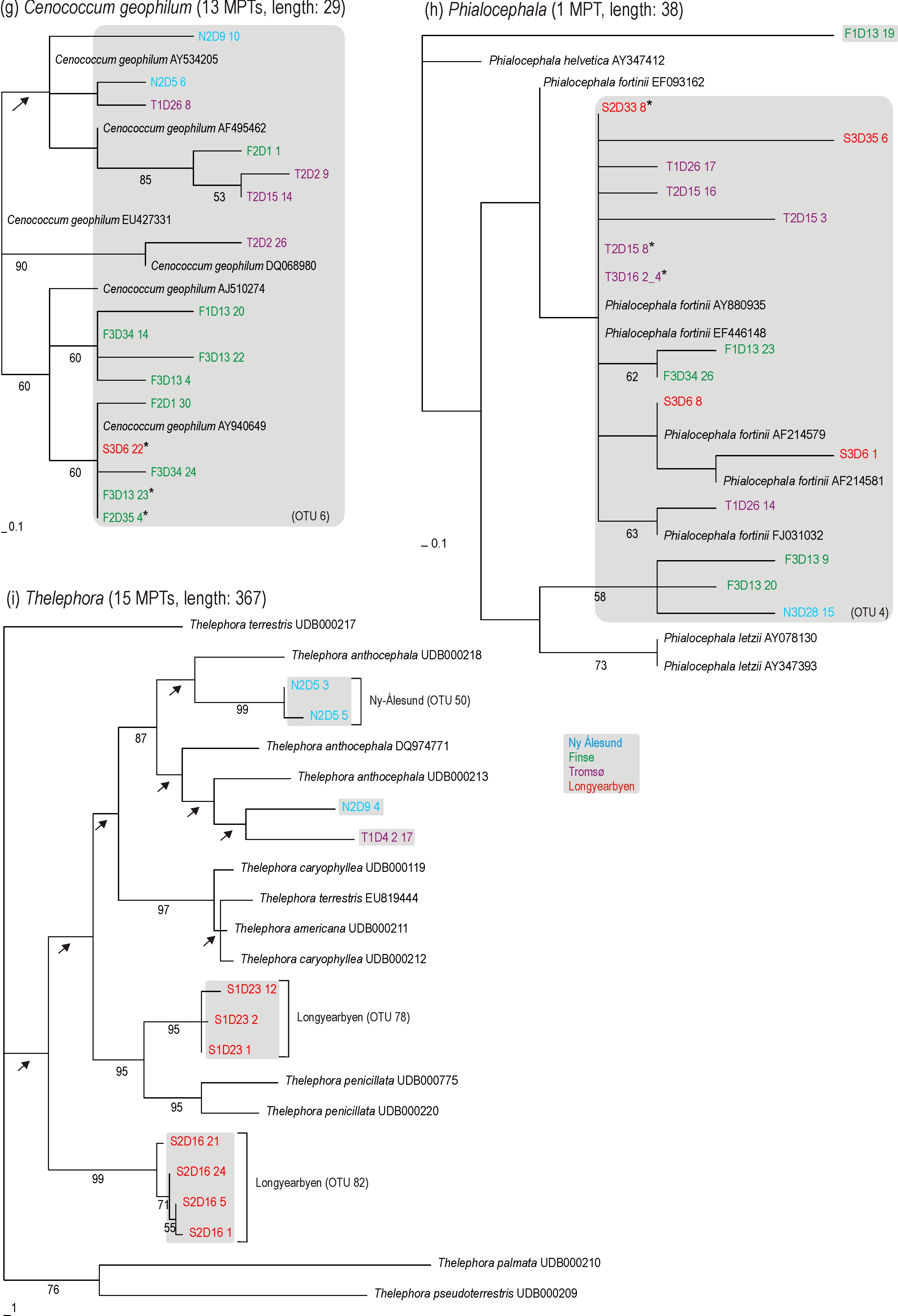

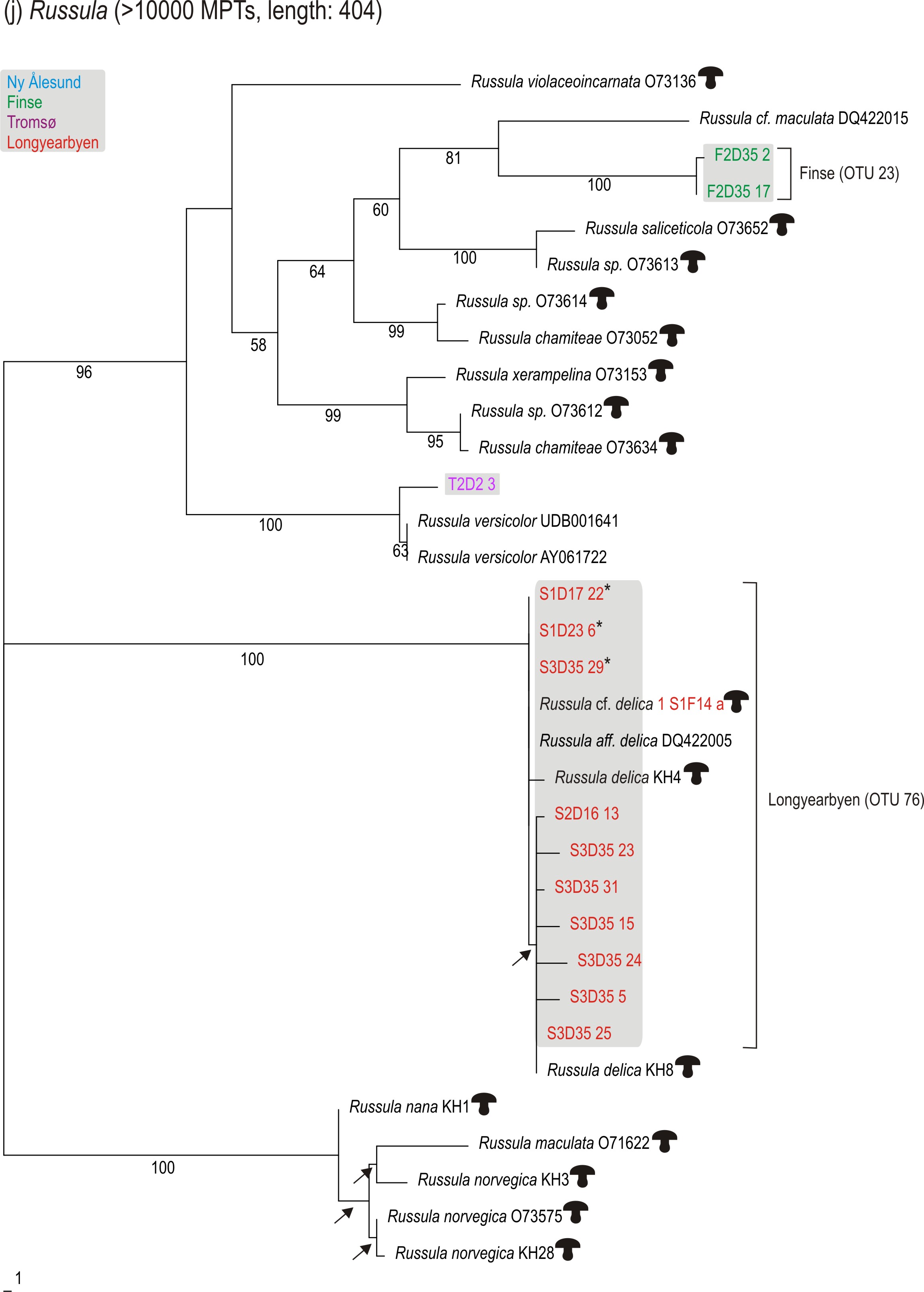

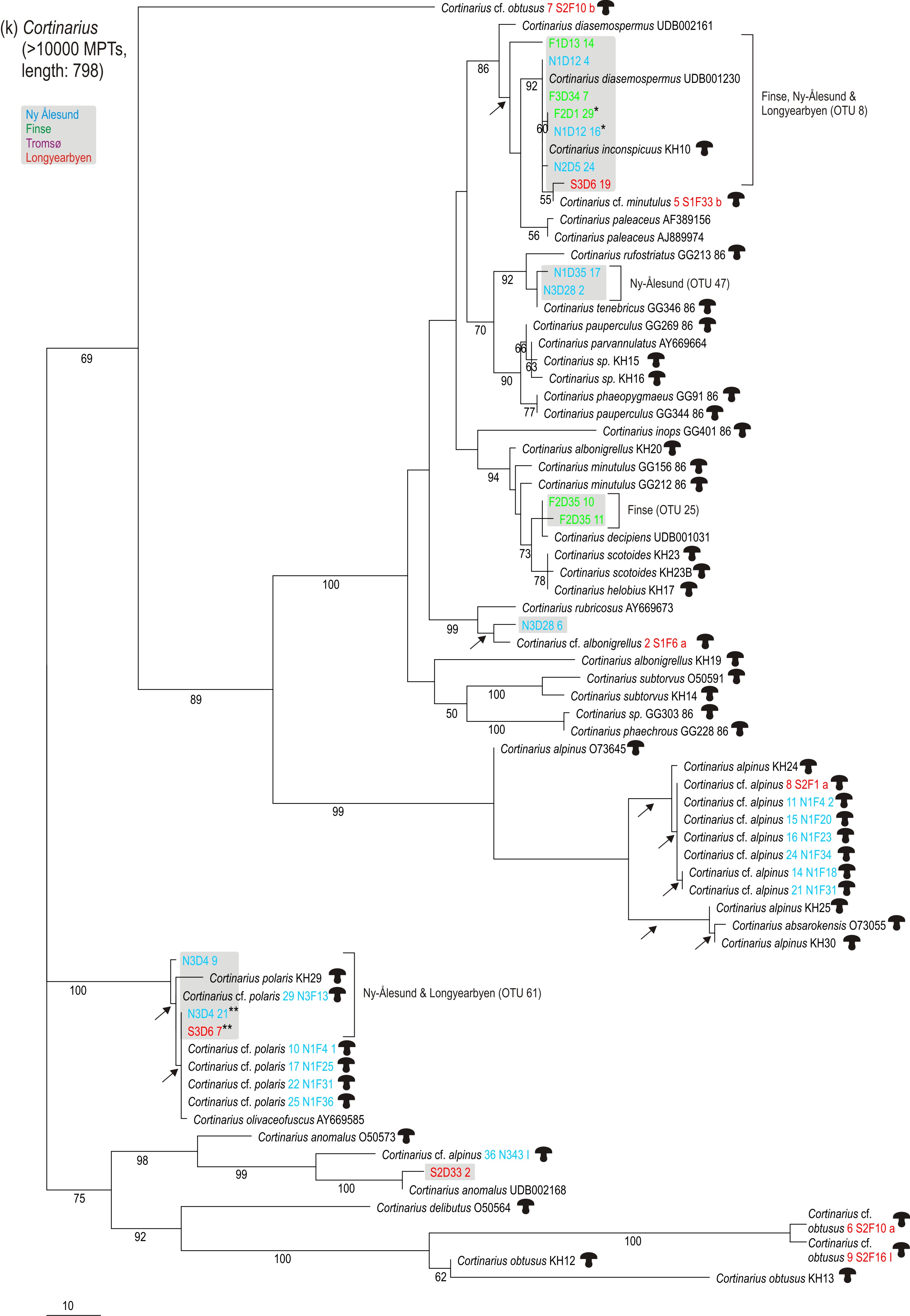

Supplement: Additional file 4 — Phylogenetic trees of 11 prevalent taxonomic groups. [file 1471-2229-10-244-S4.DOC]
