# Supplementary material for: High diversity of root associated fungi in both alpine and arctic Dryas octopetala
Source: BMC Plant Biol. 2010 Nov 11;10:244. doi: 10.1186/1471-2229-10-244 (PMC3095326; doi:10.1186/1471-2229-10-244)
Supplement: Additional file 5 — Observed and estimated number of OTUs. [file 1471-2229-10-244-S5.DOC]

Additional file 5 - Observed and estimated number of OTUs

Observed and estimated number of OTUs for the four main localities as extrapolated from the calculated species accumulation curves (see Fig. 5).

| Locality | Observed no. of OTUs^1^ | Estimated no. of OTUs^2^ | | | | | | | | | |
| --- | --- | --- | --- | --- | --- | --- | --- | --- | --- | --- | --- |
|  | 6/  144 | 1/  24 | 6/  144 | 26/  624 | 50/  1200 | 100/  2400 | 500/  12000 | 10^3^/  24×10^3^ | 10^4^/  24×10^4^ | 10^5^/  24×10^5^ | 10^6^/  24×10^6^ |
| Finse | 34 | 5 | 31 | 53 | 63 | 73 | 96 | 106 | 140 | 154 | 169 |
| Longyearbyen | 37 | 5 | 34 | 58 | 68 | 80 | 106 | 117 | 154 | 161 | 177 |
| Ny-Ålesund | 45 | 7 | 42 | 71 | 84 | 97 | 129 | 143 | 188 | 204 | 229 |
| Tromsø | 49 | 6 | 45 | 77 | 91 | 106 | 140 | 155 | 205 | 215 | 226 |

^1^Number of root systems sampled/number of clones sampled. ^2^Estimated number of root systems sampled/number of clones sampled (24 clones per root system). For example, if 100 root systems with 24 clones per root system had been sampled, 73, 80 97 and 106 OTUs, respectively, are estimated for the four main localities.
