# Supplementary material for: High diversity of root associated fungi in both alpine and arctic Dryas octopetala
Source: BMC Plant Biol. 2010 Nov 11;10:244. doi: 10.1186/1471-2229-10-244 (PMC3095326; doi:10.1186/1471-2229-10-244)
Supplement: Additional file 6 — Extrapolated total species (T-S) curves. [file 1471-2229-10-244-S6.DOC]

**
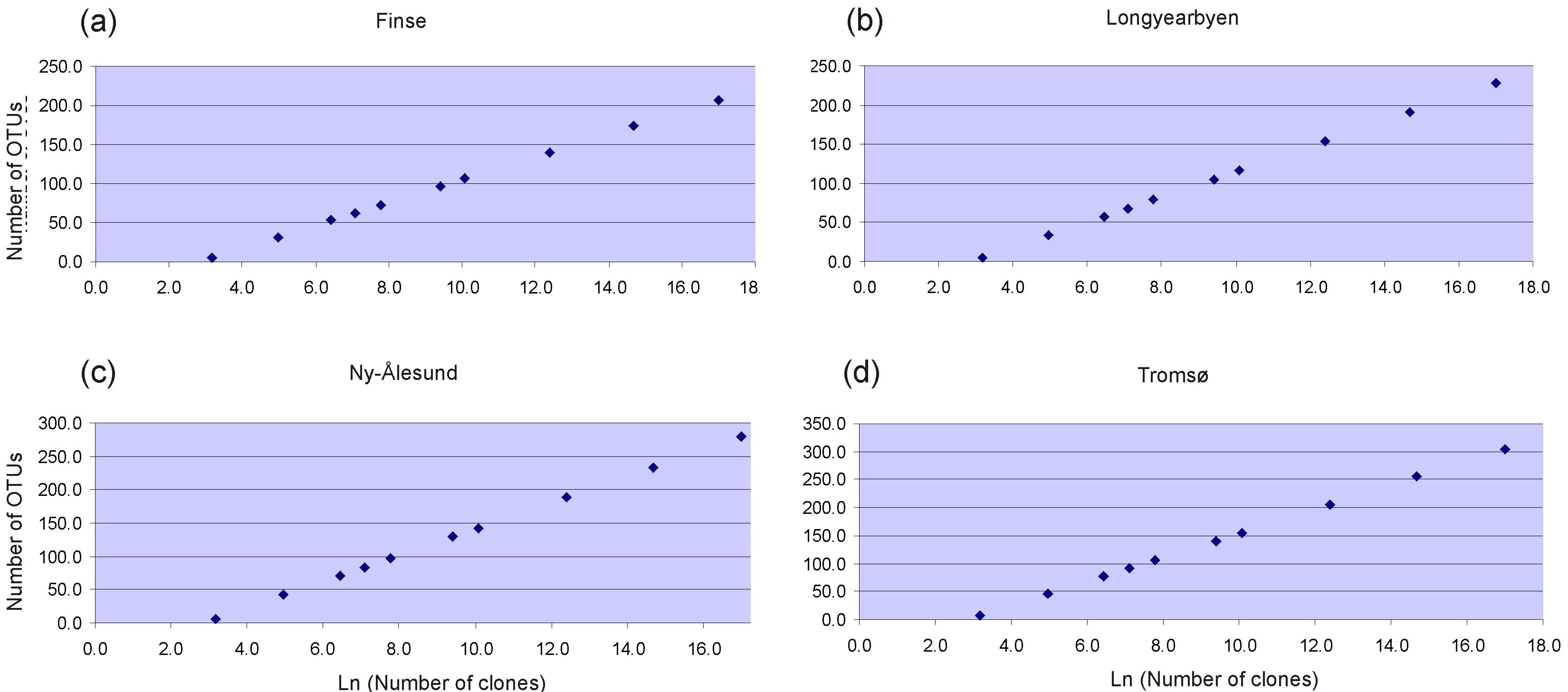
**

**Additional file 6 - extrapolated total species (T-S) curves**

A total species projection curve (T-S) is the smooth curve through the average total number of species in all combinations of the subareas in the species-accumulation curves. The rate of increase in the T-S curve is the increment in the ratio between the current species number (S) and logarithm of the number of samples (c), i.e. the rate of change of S/ln(c). As can be observed in the T-S curves for (a) Finse, (b) Longyearbyen, (c) Ny-Ålesund, and (d) Tromsø, the curves show no sign of reaching a plateau, demonstrating the high degree of heterogeneity in the fungal communities.
